# Local measurement of terahertz field-induced second harmonic generation in plasma filaments

**DOI:** 10.1007/s12200-023-00095-y

**Published:** 2023-12-13

**Authors:** Kareem J. Garriga Francis, Xi-Cheng Zhang

**Affiliations:** The Institute of Optics, University of Rochester, Rochester, NY 14627 USA

**Keywords:** Terahertz detection, Plasma characterization, Spectral characterization, Single-shot detection

## Abstract

The concept of Terahertz Field-Induced Second Harmonic (TFISH) Generation is revisited to introduce a single-shot detection scheme based on third order nonlinearities. Focused specifically on the further development of THz plasma-based sources, we begin our research by reimagining the TFISH system to serve as a direct plasma diagnostic. In this work, an optical probe beam is used to mix directly with the strong ponderomotive current associated with laser-induced ionization. A four-wave mixing (FWM) process then generates a strong second-harmonic optical wave because of the mixing of the probe beam with the nonlinear current components oscillating at THz frequencies. The observed conversion efficiency is high enough that for the first time, the TFISH signal appears visible to the human eye. We perform spectral, spatial, and temporal analysis on the detected second-harmonic frequency and show its direct relationship to the nonlinear current. Further, a method to detect incoherent and coherent THz inside plasma filaments is devised using spatio-temporal couplings. The single-shot detection configurations are theoretically described using a combination of expanded FWM models with Kostenbauder and Gaussian *Q*-matrices. We show that the retrieved temporal traces for THz radiation from single- and two-color laser-induced air-plasma sources match theoretical descriptions very well. High temporal resolution is shown with a detection bandwidth limited only by the spatial extent of the probe laser beam. Large detection bandwidth and temporal characterization is shown for THz radiation confined to under-dense plasma filaments induced by < 100 fs lasers below the relativistic intensity limit.

**Graphical Abstract:**

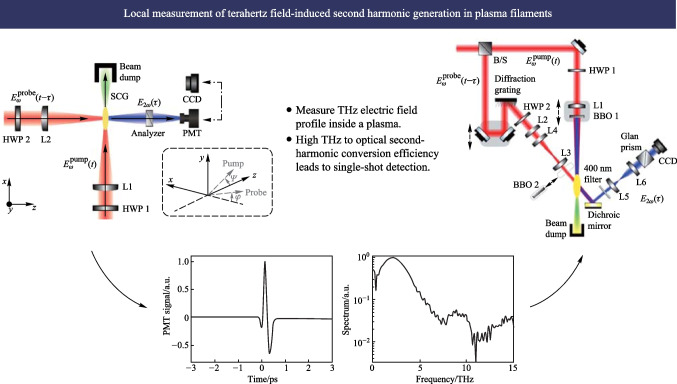

## Introduction

**Fig. 1:**
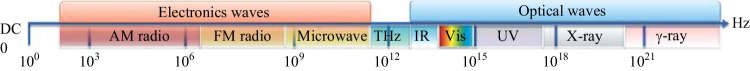
Electromagnetic spectrum. The THz region is the intersection between electronic and optical waves

The terahertz (THz) band is a rather elusive region in the electromagnetic (EM) spectrum. Informally defined as a region between 0.1 and 10 THz (30 μm to 3 mm), this region is also known as the mm wave region and is shown in Fig. 1. The fact that at the time of this writing, there are not many efficient sources nor emitters—or at least coherent ones—within this band contributes to this section once being designated as the “THz gap” [1, 2]. This band is an overlap of the far infrared (IR) wave band (wavelengths from 15 μm to 1 mm) and the microwave band (wavelengths from 1 mm to 1 m).

Because the photon energy at THz frequencies is low (in meV range), the application of THz waves in biomedical imaging and sensing has enjoyed great success. The photons are nonionizing and can be used to nondestructively analyze samples. Some applications in the biomedical sector include cancer detection/screening [3–7], food/pharmaceutical quality control [8–11], and behavioral analysis [12–14]. The nonionizing property has also been exploited for explosives detection and restoration of historical records [1].

The development of high peak power and ultra-short-pulsed lasers has accelerated the closing of the THz gap and issued a new directive with the promise of “extreme THz science” [15–19]. Chief within this directive is the desire to develop table-top THz sources reaching peak electric field strengths of GV/cm and energies exceeding the mJ-level. With these sources, breakthroughs are expected in electron accelerators since THz sources can provide higher acceleration gradients through much shorter distances when compared to conventional radiofrequency (RF) accelerators and much better power scaling capabilities when compared to laser wake-field accelerators [20–23]. Further, sources developed via the extreme THz science directive will enable researchers to exploit material nonlinearities in the THz regime. As such, the development of THz filamentation [24, 25], soliton propagation [26], harmonic generation [27–29], electromagnetically induced transparency [30], etc., promise a bright future for the field. Plasma-based sources have been at the forefront of the development of bright THz sources because they provide broadband and high-intensity methods of THz generation [18, 31–35].

There are two dominant technologies in the fully coherent detection of THz radiation: electro-optic sampling (EOS) [36] and THz nonlinear third-order detection [37–39]. EOS is a second-order nonlinear detection of moderated THz field. In this document, we do not consider the photo-conductive antenna methods [1, 2, 40] as they are not typically used in systems with high pump laser energy. The method of EOS is a pump–probe technique that uses the nonlinear electro-optic (EO) coefficient in nonlinear crystals to correlate changes in optical probe polarization to changes in the electric field evolution of the THz inside the crystal [1, 36, 41]. This method is limited by pulse duration, mismatch between pump group velocity and THz phase velocity, and dispersion. As such, the crystals used for detection must be kept thin because as their thickness increases, the sensitivity increases, but the velocity mismatch also worsens [2]. The advantage of EOS is in procuring phase information with shot-noise limited performance, but the system is complex and is limited in detection bandwidth by nonlinear crystal phonon absorption resonances.

Because the optical-THz conversion efficiencies are often low for materials investigated under the extreme THz directive (< 7%) [18, 42–44], and because large-scale laser facilities with low repetition rate lasers are often used to provide the most energetic optical pulses, there is also a need to determine THz spectral content and waveforms within a single laser shot or pulse. Single-shot detection methods have emerged in the form of various EOS schemes [45–50]. Most single-shot EOS systems depend on directly coupling temporal information onto the spatial extent of the beam. This is typically done by exploiting spatio-temporal coupling (STC), which are optical design methods where $E(x,y,z;t) \ne E(x,y,z) E(t)$ [51, 52]. Coupled with the growing interest to investigate THz nonlinear effects, single-shot techniques also offer a chance to observe non-reversible effects due to THz radiation [53]. While single-shot EOS systems have their advantages, they are still marred by the same issues present in their multi-shot counterparts. More present is the crystal over rotation issue: when a THz electric field is strong enough to induce a π/2 phase-shift birefringence in EOS, there is an ambiguity and distortion of the detected THz field. This makes determining the correct electric field strength and profile difficult [1, 2, 41]. Additionally, the spectral and temporal resolution tend to suffer.

In addition to EOS which relies on second order (electric field-dependent) nonlinearity in crystals, third order (intensity dependent) nonlinearities can also be exploited [30]. In general, the inverse four-wave mixing (FWM) process used to phenomenologically describe two-color air plasma [33] can be used to describe the appearance of an SH of the probe when in the presence of THz via the expression:

1 $${S}_{2\omega } \propto {\int }_{-\infty }^{\infty }{\left|{\chi }_{ijkl}^{\left(3\right)}{E}_{\omega }\left(t - \tau \right){E}_{\omega }\left(t - \tau \right){E}_{\mathrm{THz}}^{*}\left(t\right)\right|}^{2}\partial t,$$

where a process ${\chi }^{\left(3\right)}\left(2\omega = \omega + \omega \pm {\omega }_{\mathrm{THz}}\right)$ can be enacted [37, 54]. Here, where ${E}_{\mathrm{THz}}(t)$, ${E}_{2\omega }(t)$, and ${E}_{\omega }(t)$ respectively represent the electric field of the THz, SH, and *ω* fundamental pulses while *χ*^(3)^ is the nonlinear third order susceptibility tensor. The nonlinear process is referred to as a THz field-induced second harmonic (TFISH) generation, which is itself a subset of a process known as electric field-induced second harmonic (EFISH) generation [55–63]. The expression in Eq. (1) is a cross-correlation integral and only leads to generation of the probe SH when there is a temporal gating occurring due to the THz field. However, because the signal is proportional only to the THz intensity, it is considered an incoherent process.

**Fig. 2:**
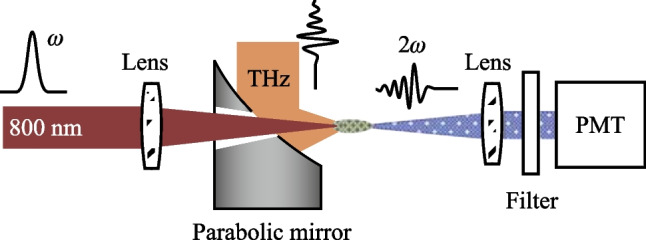
A conventional THz-ABCD system as presented in Ref. [54]. A two-color air plasma THz generation system is used to produce a THz wave. A second plasma created by the probe beam is used as a local oscillator to detect the THz field/intensity

Because ${\chi }^{\left(3\right)}$ is present in all materials, the TFISH system is more robust than EOS and can be enacted in gases [37], liquids [39], centrosymmetric solids [38, 64], and noncentrosymmetric solids [65, 66]. A generalized method for coherent (electric field dependent) detection using TFISH involves combining the SH signal with an independently controlled SH signal—termed here as a local field-induced second harmonic (LFISH). The interference between both SH signals, $$\begin{array}{c}{S}_{2\omega } \propto {\left|{E}_{2\omega }^{\mathrm{LFISH}} + {E}_{2\omega }^{\mathrm{TFISH}}\right|}^{2},\end{array}$$ leads to the appearance of three SH sources: (1) the conventional TFISH, (2) the conventional LFISH, and (3) a cross-term between LFISH and TFISH that depends on the THz field rather than the intensity [37, 54]. The most popular version of the third order nonlinear detection is the air-biased coherent detection (ABCD) system shown in Fig. 2 and introduced by Dai et al. in 2006 [37]. The nonlinear detection scheme has been shown in gases by using either a secondary air plasma or a bias electric field as an LFISH, in liquids by using a β-BBO produced SH as an LFISH [39, 54, 67, 68], and in centrosymmetric solids by using bias fields as LFISH sources [38]. Compared to EOS, this technique boasts a much larger detection bandwidth (limited on the order of the inverse of the optical probe pulse duration) and no phonon absorption/over-rotation issues. Unfortunately, the method has a lower SNR compared to EOS and requires a high input electric field [69, 70]. A potential reason for this may be due to intensity and phase fluctuation caused by the appearance of plasma at the focal region.

The THz landscape is changing, and the THz gap is quickly closing with the rapid development of higher peak electric field and high energy THz sources [18, 44, 71, 72]. From the previous sections, the limitations of EOS are clear and unfortunately, a TFISH or coherent nonlinear detection system that operates in real-time or single shot does not currently exist. One of the main reasons for the absence of a single-shot TFISH system is that the third order nonlinear process either requires very high optical intensity, or very high peak THz electric field via Eq. (1). Unfortunately, there is a limit to the intensity the optical probe can reach through the filament intensity clamping effect [25, 73–77]. After reaching a probe intensity of 1 × 10^14^ W/cm^2^, the residual energy only contributes to the filament reservoir or to multi-filamentation effects and pulse splitting. Because of that, the second harmonic produced will be saturated. Because the THz is non-ionizing, and because it is always preferable to work with lower optical intensities, it is better to find a way to increase the THz peak electric field. However, current facilities that can produce high electric field sources only operate at either low repetition rates or explicit single shot modalities. This makes creating a test platform for single-shot detection using TFISH very difficult.

With growing interest in new areas of optics taking advantage of STCs rather than trying to mitigate them [78–83], there are also new opportunities in improving the designs of the single shot detection systems and transplanting them to TFISH. In this paper, a new method of analyzing plasma systems is developed to directly analyze THz in the plasma filament. The advantage of this method is access to a much larger peak electric field due to the extreme spatial confinement of THz by the filament.

## Terahertz field induced second harmonic generation at 90° incidence

The focus of this section is the presentation of the noncollinear terahertz (THz) evaluation system used throughout this work. The THz field-induced second harmonic (TFISH) generation model, a four-wave mixing (FWM) process, is used to explain the results obtained. The approach and motivation for analyzing the TFISH noncollinearly and in the region of the plasma is first discussed. Next, an experimental overview and description is presented along with data obtained from the system.

### Experimental motivation

One of the major issues hindering the development of a single-shot TFISH system—as discussed in the previous section—is the lack of availability for a test platform in the systems that require single-shot analysis. The fact that intensity clamping prevents air-based systems from using larger pulse intensity and the fact that the TFISH process efficiency is very low make the development of a single-shot TFISH very difficult. In a conventional TFISH system, the energy conversion efficiency from optical fundamental probe to optical second harmonic (SH) is very low on the order of ${10}^{-9}$ % [37, 39]. Recent work by Tan et al. has shown that the conversion efficiency can be significantly improved by one order of magnitude if instead of using air, a condensed matter was used [39]. Using water, for example, the threshold optical probe needed could also be reduced significantly because the third order susceptibility of liquids is much larger than that of gases [26, 30]. Unfortunately, the intensity clamping effects persist in liquids and the TFISH process remains limited.

**Fig. 3:**
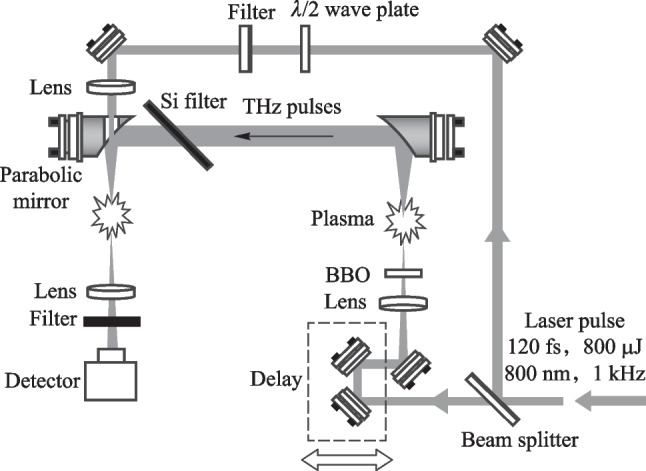
A conventional TFISH system as presented in Ref. [37]. The system is a Mach–Zehnder interferometer

In general, a TFISH system is built in an interferometer setup using imaging techniques to guide the generated THz from the source plane to the detector plane. As seen in Fig. 3, a conventional system is built as a Mach–Zehnder interferometer [84] where an input optical beam is split between a pump and probe by a beam splitter. The pump beam is used to generate the THz while the probe beam is used to perform a cross-correlation with the THz [85]. In the TFISH case, the guiding of THz from the source plane to the detection plane typically happens with parabolic mirrors due to their achromaticity and lack of spherical aberration. Most TFISH systems will use a two-color air plasma-based source because it provides the highest available THz electric field strengths considering the 800 nm optical pumping that is widely commercially available.

The motivation behind the work in this paper lies behind the recognition that because all TFISH systems exist on a platform of imaging systems, the THz electric field at the source will always represent the strongest electric field components. Measurements of the THz field in the plasma have historically proven quite difficult considering that the ionization occurs above the damage threshold of any detector that could be used. Fortunately, the TFISH method is non-contact and so long as the plasma is under-dense, the optical frequencies propagate and can be used to characterize the source [86]. Additionally, optical filaments have been known to show very strong spatial confinement. This means that the THz field strengths available at the filaments must be many orders of magnitude larger than their detector plane counterparts because the THz beam spot size is reduced significantly. This last point was initially shown by Zhao et al. in 2016 where using a knife-edge test, it was suggested that the THz source at the filament was strongly confined to a few microns [87].

#### Issues in the plasma system

An under-dense plasma is unfortunately not an ideal detection plane for THz because of its high nonlinearity and the plethora of linear and nonlinear frequency generation phenomena [24]. When seeking the TFISH signal, several other SH or near SH sources of radiation exist in the plasma.

**Fig. 4:**
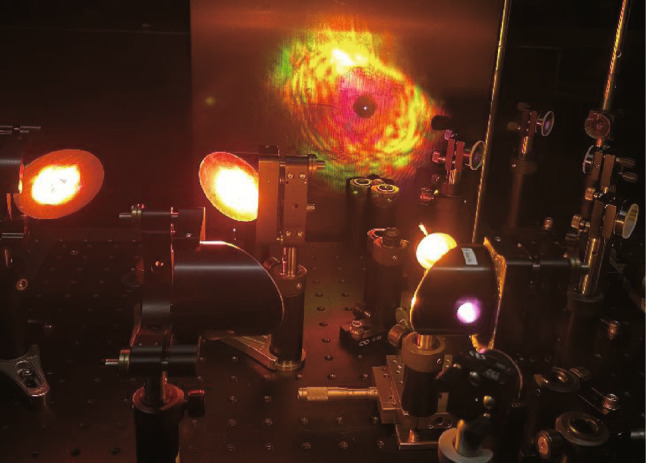
Image of a plasma supercontinuum taken in a two-color air plasma THz system

The first and most obvious source of near SH signals is the supercontinuum from the plasma [25] (shown in Fig. 4). The cause of supercontinuum is the strong spectral broadening due to the formation of the filament. Because of the intensity dependent index modification in the filament, a femtosecond beam will modify its own phase during propagation in a process known as Kerr self-phase modulation (SPM) [26, 30]. The SPM process is typically accompanied by Raman effects and higher-order nonlinear phenomena such as self-steepening that serve to add more frequencies to the spectrum [24, 25]. The radiation caused by the supercontinuum is constrained to the forward direction following the Poynting vector of the pulse. To avoid collecting the supercontinuum, the noncollinear system impinges a probe away from the propagation direction.

In addition to supercontinuum, radiation near the 800 nm SH will be generated due to ionized air glow and air lasing transitions. In the first case, the recombination process in the plasma will allow for the radiation of frequencies corresponding to the gas species spectral lines [24, 25, 88]. The strong lines of nitrogen occur near 400 nm and due to the high temperature of the electron gas plasma, they can appear to be quite broad. This makes it difficult to separate true plasma SH from the ionized air glow. Fortunately, with a pump–probe system, the ionized air glow can be discounted by using a spectrometer to measure SH signal with and without a probe beam. If the SH is still present in the absence of a probe beam, the radiation observed is likely ionized air glow and not true SH.

External pumping from a secondary probe can also lead to the generation of lasing transitions. First, the presence of a plasma causes an amplified spontaneous emission process (ASE) which leads to radiation of < 400 nm radiation in nitrogen gas. The radiation is shown to have an exponential dependence on pump laser energy and is characteristically unpolarized as discussed in Ref. [89]. Second, the spectral broadening of the fundamental frequency caused by the plasma produces frequencies in the range of the nitrogen lines that can act as seeding pulses for lasing action. As shown in Refs. [90–94], the transitions are characteristically narrow and radiate with a polarization parallel to the ionizing pulse.

Lastly, strong SH can be generated due to the ponderomotive force itself [57, 95–107]. In a nonuniform plasma, the effective second order polarization takes a form:

2 $${P}^{\left(2\right)}\left(2\omega \right)\propto {\chi }_{\mathrm{eff}}^{\left(2\right)}\left[a{\nabla }^{2}\left(\overrightarrow{ E}\cdot \overrightarrow{E}\right) + b\left( \overrightarrow{E}\cdot \nabla {N}_{\mathrm{e}}\right)\overrightarrow{E}\right],$$

where *P*^(2)^ denotes the second-order polarization, *χ*_eff_^(2)^ is an effective second-order susceptibility tensor with strong dependence on the electron number density, *a* and *b* are proportionality constants, and $\nabla$*N*_e_ is the gradient of the plasma density. The first term is a gradient and produces an irrotational polarization. This term cannot radiate in bulk plasma and is therefore washed out by the second term which leads to radiation parallel to the optical electric field [57, 99, 107]. For linearly polarized optical pulses, since the plasma gradient is cylindrically symmetric and perpendicular to the laser propagation, the expression in Eq. (2) generates radiation in two lobes oriented along the azimuth of the polarization. Unfortunately, even though the SH generated in this manner is a nonlinear optical signal, a power dependence experiment cannot be used to segregate the effects from SH due to TFISH/ EFISH. This is because both methods produce a signal that is dependent on the intensity of the optical beam [30, 57]. However, because the plasma lifetime in typical THz experiments exceeds the duration of the pulse, and because in a pump-probe system there is not a gating mechanism in Eq. (2), the SH generated in this manner in the temporal regime will be seen to decay as a function of *N*_e_(*z*,*t*). Therefore, under normal circumstances, the beam shape, polarization, and temporal dynamics can be used to discount this radiation due to pure *χ*^(2)^ effects.

#### Accounting for TFISH and EFISH

An under-dense plasma has the capability to produce an SH signal from either a TFISH, EFISH, or joint process. In general, a charge separation is induced which leads to an electric field. An analysis of the maximum values that the induced electric field can reach was done by Bethune in 1981 while analyzing the results of Miyazaki et al. regarding EFISH generation in atomic vapor from 1.06 μm ps lasers [57, 100]. In his publication, Bethune states that the maximum value of the induced electric field in an EFISH system will depend on the shortest of the following characteristic times [57]: (1) the 1/e pulse duration of the laser, ${T}_{\mathrm{o}} = \frac{{\tau }_{\mathrm{FWHM}}}{2\sqrt{\mathrm{ln}(2)}}$, where *τ*_FWHM_ is the full width at half maximum (FWHM) pulse duration; (2) the inverse plasma frequency *T*_p_; (3) the time it takes to move a singly ionized electron a distance of one focal spot radius, ${T}_{\mathrm{f} }\approx \sqrt{\frac{2 m {W}_{\mathrm{f}}}{{F}_{\mathrm{p}}}}$, where *W*_f_ is the focal radius and *F*_p_ is the time-averaged ponderomotive force felt by the electron; and (4) the time it takes an electron with some initial kinetic energy *U*_*v*_ to escape the beam axis, ${T}_{v }= \frac{{W}_{\mathrm{f}}}{v}$, where* v* is the electron velocity.

In an under-dense plasma, the electrons cannot be accelerated relativistically or near relativistically. Given that the ponderomotive velocity plays the key role in the electron acceleration process, case (4) above is neglected since *T*_*v*_ is the largest time although the maximum electric field from this process is the largest [57]. Next, considering that the excitation intensity is non-relativistic (*I* < 10^18^ W/cm^2^), *T*_f_ remains above ps regime, so it is neglected. As such, only the presence of charge separation limited by *T*_p_ and *T*_o_ is considered. Bethune’s work predates the discovery of THz radiation by Hamster and Falcone in 1990 [31]. The typical electron plasma density in an under-dense plasma THz experiment typically spans the range ${10}^{14} \frac{1}{{\mathrm{cm}}^{3}} \le {N}_{\mathrm{e}} \le {10}^{18} \frac{1}{{\mathrm{cm}}^{3}}$, yielding a value of $40 \, \mathrm{fs} \le {T}_{\mathrm{p}} \le 4\, \mathrm{ps}$ and a maximum achievable charge separation-induced field of 1 MV/cm when an electron velocity of 0.002*c is considered with 800 nm pumping [108–111]. In a THz experiment, 1–10 MV/cm peak electric field strengths are attainable while focusing the THz to mm sizes in two-color experiments. This means that if spatial confinement of the THz to a size near a few μm is possible, the maximum electric field could register an increase in the THz peak field over two orders of magnitude.

### Experimental setup

**Fig. 5:**
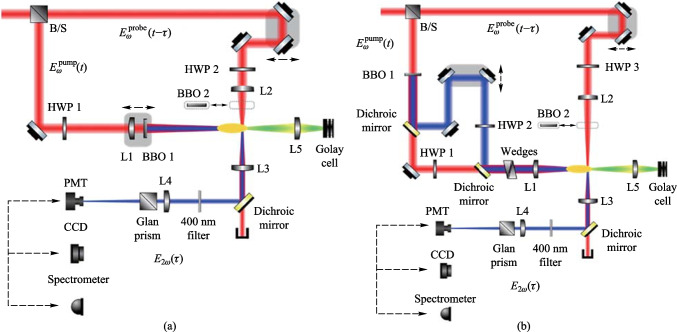
System schematic for the noncollinear TFISH system at 90° analysis. The system is a two-color air plasma THz generation system and embeds two subsystems: **a** case 1: a β-BBO (BBO 1) is placed after Lens L1 and is translated toward the focus to control the phase between pump *ω* and 2*ω*; and **b** case 2: a Mach–Zehnder phase compensator is used to control the phase between pump *ω* and 2*ω*. For both subsystems, a second β-BBO (BBO 2) can be used along the probe beam to procure a coherent signal via interferometry

Figure 5 shows the experimental setup for the 90° noncollinear TFISH detection system. A 6.5 W and 1 kHz amplified laser system [Coherent Astrella] operating near 100 fs is used. The laser fundamental wavelength is 800 nm, and the initial beam spot size is measured to be 12 mm 1/e^2^ by knife-edge test. A beam splitter is used to separate the beam onto pump (plasma excitation) and probe (SH detection) arms. A delay can be added either to the pump or probe to control the timing between both beams. The pump in the system has a maximum average power of 3 W (3 mJ). The two-color excitation exists in two subsystems: case 1 in Fig. 5a has a 100 μm thick type I β-barium borate (BBO) crystal placed between the pump lens (lens 1) and the plasma while case 2 in Fig. 5b has the same BBO crystal moved before the lens 1 as part of a phase compensator to separately control the *ω* (800 nm) and 2*ω* (400 nm) fields generated by the BBO. In case 1, the pump *ω* polarization is controlled with a half-waveplate (HWP). The 2*ω* pump polarization is controlled by rotating the crystal along the azimuth (around the propagation axis). The BBO is rotated until the *ω* polarization becomes elliptical with its major axis having components along the extraordinary axis (approximately 30° from the position where the *ω* polarization matches the ordinary axis) to maximize the far-field THz signal.

In case 2, shown in Fig. 5b, a set of HWPs along the *ω* and 2*ω* paths allow for independent control of polarization for all fields. This sub-system also has a Mach–Zehnder phase compensator to control the relative phase between the pump frequencies prior to focusing [112]. The paths are frequency discriminated by using a combination of dichroic mirrors and bandpass filters for the respective frequencies. The 2*ω* pump delay in Fig. 5b can be scanned independently for a course adjustment of timing between the two pumps while the wedges are used for fine adjustment.

Crucially, both subsystems have their pump lenses placed on a mechanical stage to allow a scan along the length of the pump plasma. This plasma is formed in atmospheric air with a 200 mm singlet lens in case 1, and a 125 mm achromat in case 2 to curb chromatic aberration. Additionally, the interaction between the pump and probe is localized to a single spatio-temporal point which yields a more sensitive TFISH signal. Single-color pump excitation scans are made by simply removing the BBO 1 crystal prior to pump focusing. The probe arms for both subsystems are the same. The probe power is never increased beyond 1 W (high power probe) and is typically kept below 25 mW for all TFISH experiments. A HWP in the probe path controls the polarization of the probe beam between s- and p-polarization. The probe beam is focused to spatially overlap with the pump beam plasma with a 150 mm focal length lens (lens L2) in both cases. A 4F imaging telescope composed of a 75 mm lens and a 125 mm lens that directly images the probe-plasma interaction onto a calibrated photo-multiplier tube (PMT) is used as the optical detection. A dichroic mirror and a set of bandpass filters (40 nm centered about 400 nm) are used to discriminate the 2*ω* TFISH signal from the ω probe. Lastly, a Golay cell is added in a 2F imaging configuration aligned along the pump pulse propagation to compare the phase modulation of the 2*ω* signal to that of the far-field THz energy [112].

#### Second harmonic spectrum

**Fig. 6:**
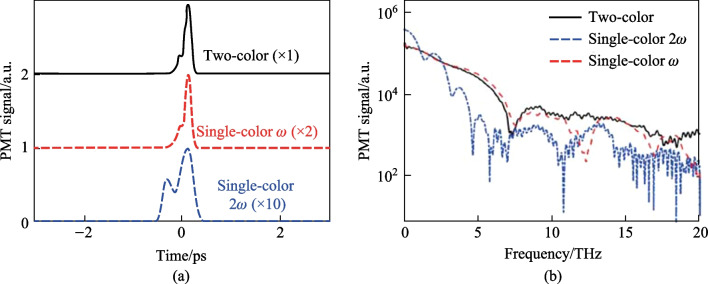
**a** Temporal TFISH signals gathered for two-color (solid line), single-color *ω* (dashed line), and single-color 2*ω* (dash-dot line) pumps. The waveforms are vertically shifted for clarity. **b** Spectra corresponding to the TFISH signals. Notably, the 2*ω* pumped TFISH bandwidth is weakest given the lower number density in the plasma. Figures taken from Ref. [113]

After initial alignment, a strong SH signal is noticed collinear to the probe beam. This signal is dependent on the pump plasma and the SH can be seen in a bright room environment. In Fig. 6a, the temporal TFISH traces are shown measured by the PMT. In case 2, by blocking the *ω* pump, the 2*ω* pump plasma can also lead to the generation of a TFISH signal, albeit a much weaker signal > 1 orders weaker in magnitude than the two-color source. The two-color plasma, single-color *ω* plasma, and single-color 2*ω* pumps were probed with 20, 100, and 500 mW probe power, respectively.

Because the TFISH signal is maintained regardless of the pump excitation scheme while only an *ω* probe is used, and because a bandpass filter is used to select only the SH of the probe, the effects of plasma scattering and fluorescence can be neglected in explaining the SH signal [113, 114]. The appearance of temporally gated signals is also encouraging in eliminating the plasma *χ*^(2)^ contributions from the system. This is because in the absence of a correlation mechanism, as the probe beam is scanned as a function of delay in Eq. (2), the SH signal should slowly decay since *N*_e_(*z*,*t*) is a slowly decaying function of time as shown in Ref. [101], and in the THz double-pump experiments in Refs. [115–120]. In the scheme of the plasma itself causing an SH signal, since the plasma lifetime is on the order of a few picoseconds, the SH should persist for a few ps. Additionally, the envelope of the SH would not have a Gaussian profile like the pulse. Lastly, in Fig. 6b the corresponding spectrum calculated by Fourier transform for the TFISH signals is shown for the three pump considerations. As expected, the spectrum is broadband with the two-color system generating the most bandwidth while the weaker 2*ω* plasma-pumped system generates the least bandwidth.

**Fig. 7:**
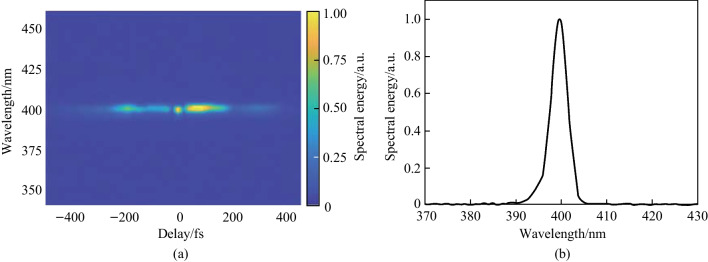
**a** Optical TFISH spectrum gathered as a function of temporal delay between pump and probe. This plot showcases that the TFISH spectrum remains consistent across time delay. **b** Spectral slice of **a** at zero delay (*τ* = 0). Figures taken from Ref. [113]

The optical spectrum of the TFISH signal produced using a two-color plasma as a function of optical delay is gathered by placing an Ocean Optics spectrometer in place of the PMT and the result is shown in Fig. 7a. For this measurement, the bandpass filters were replaced with a single bandpass filter that passed radiation between 300 and 700 nm. The 800 nm peak of the fundamental probe is missing because the dichroic mirrors do not effectively reflect 800 nm. The amplitude of the spectral energy follows the envelope of the TFISH trace shown in Fig. 7a. A slice of this spectrum measured at delay *τ* = 0 is shown in Fig. 7b and yields an ideal Gaussian spectrum closely following the expectation of second-harmonic generation (SHG) of the probe spectrum. When examining the spectrum of the SH field, an absence of peaks or features away from 400 nm—especially the absence of sharp peaks along 350 nm—is used to eliminate the effects of air-lasing [90] and THz radiation-enhanced emission of fluorescence (REEF) [121] as an explanation for the bright blue light observed. The signal energy is measured to be 3.7 μW for a two-color source with a sensitive power meter when *a* < 20 mW probe energy is used, leading to a probe to SH conversion efficiency > 0.02% when a two-color pump is used. This value is nearly two orders stronger than that observed from SHG induced via plasma asymmetry in air even when spatio-temporally altered laser fields are used to increase the effect of symmetry breaking [97, 122]. It is also five orders higher than typical TFISH experiments [39]. The corresponding conversion efficiency for single-color *ω* pumping and 2*ω* pumping was found to be $4\times {10}^{-4}$% and $2\times {10}^{-4}$%, respectively. Note that when the probe or pump is independently blocked, the SH signal disappears.

#### Plasma scan

**Fig. 8:**
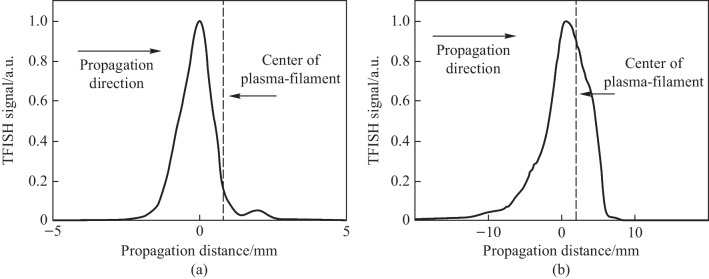
Typical plasma scan (P-scan) plots produced by scanning the probe along the plasma length while maintaining the same timing between pump and probe in system case 1 for **a** single-color* ω* pumping and **b** two-color pumping. The zero position indicates the position of the strongest TFISH, the dashed line shows the estimated center of the plasma filament, and the arrow shows the laser propagation direction. Figures taken from Ref. [113]

As mentioned in the experimental setup, a plasma scan is performed by shifting the pump lens through the focus to show how the TFISH signal depends on the positional overlap within the plasma. Figure 5a shows the lens L1 on a translation stage that is used to shift the plasma position. Since in plasma, *N*_e_ is a function of time and positional coordinate *z*, and because the THz will depend on *N*_e_, it is important to find the optimum position to overlap the probe beam. In Fig. 8a the data shows the plasma scan (P-scan) for a single-color *ω* pumped signal while (b) shows the P-scan trace for a two-color pumped signal. Zero denotes the peak of the plasma scan plot and does not coincide with the brightest section of the visible plasma filament nor the geometrical focus. This may be because the higher electron number density in this region leads to some absorption of the THz signal and decreased 2*ω* signal. Beyond the peak point, the TFISH signal quickly decreases likely due to a divergence of the THz wave when exiting the filament. The plasma scan plot spans a length larger than the visible light spark and spans a length much larger than the pump laser’s Rayleigh length. Because of this, the plasma scan uncovers some of the plasma “invisible length”.

**Fig. 9:**
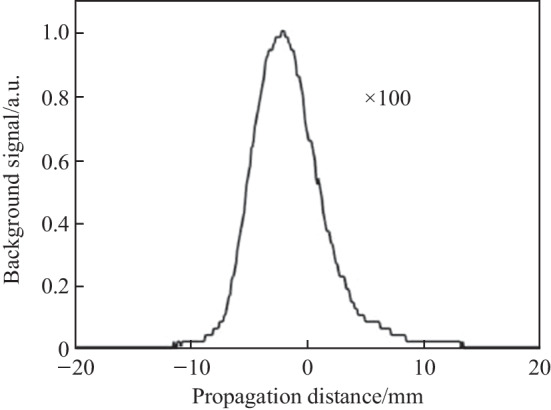
Background plasma fluorescence signal retrieved by performing a P-scan while blocking the probe. The 100 × indicates that the fluorescence peak is 100 × weaker than the signal when the probe is unblocked

When the probe beam is blocked, a P-scan reveals the scattered fluorescence near 400 nm that arrives at the detector as shown in Fig. 9. However, a scan of the main delay between pump and probe does not reveal any temporal signals. The magnitude of the P-scan trace signal when the probe is blocked is equivalent to the magnitude of the noise when the probe beam is unblocked. As such, plasma fluorescence is the major limiting noise source contributing to the noncollinear TFISH measurement.

#### Visible SH signal

**Fig. 10:**
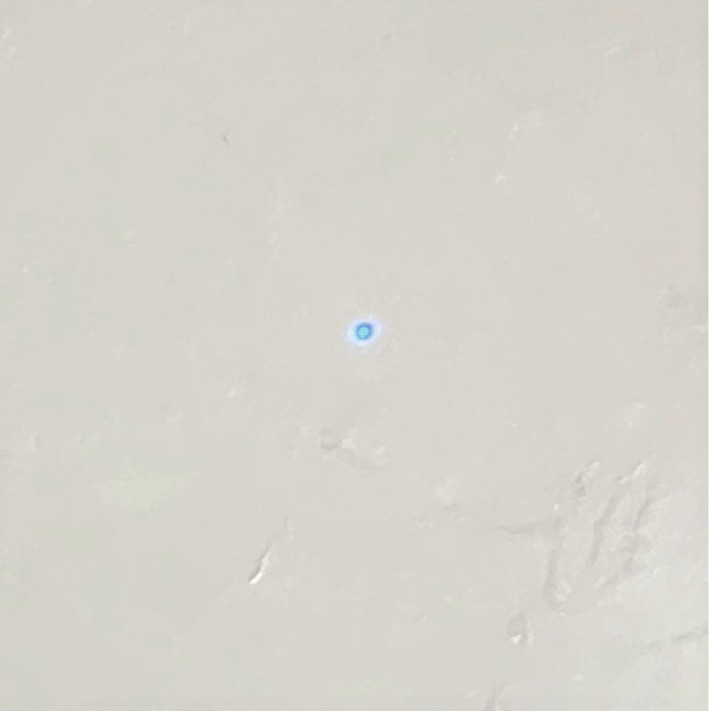
Image of the focal spot of the TFISH signal captured with a cellphone camera. Image taken from Ref. [113]

When the PMT in Fig. 5 is removed and replaced with a white notecard, Fig. 10 shows the focus of the final lens in the 4F imaging system onto a white notecard while the lights are on in the laboratory. The result shown is for a fully optimized two-color pumped TFISH system with < 20 mW probe beam. A bright SH signal can be easily seen. The image was captured with a cellphone camera.

#### Power dependence and Gaussian divergence

**Fig. 11:**
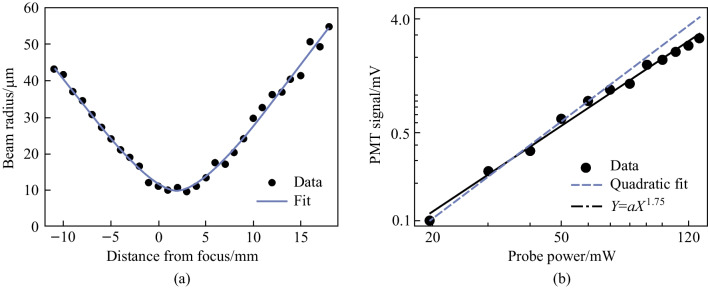
**a** Plot of the beam divergence for a TFISH signal as a detector is scanned through the focus of the 4F imager. A Gaussian divergence fit shows that the TFISH signal is indeed phase-matched to the optical probe beam. **b** Dependence of the TFISH signal on probe Energy plotted on a double log scale. Figures adapted from Ref. [113]

The TFISH signal has a Gaussian beam divergence as shown in Fig. 11a. The PMT is replaced with a CCD to record various TFISH signals as a function of position away from the focus of the TFISH signal. Setting *z* = 0 as the best focus position of the TFISH, the 1/e^2^ radius of the beam is then calculated to show the propagation of the beam waist along the focal region. A fit to the Gaussian waist expression $W\left(z\right)={W}_{\mathrm{o}}\sqrt{1+{\left(\frac{z}{{z}_{\mathrm{R}}}\right)}^{2}}$, where *W*_o_ is the minimum beam waist radius and ${z}_{\mathrm{R}}= \frac{\uppi n {{W}_{\mathrm{o}}}^{2}}{\lambda }$ is the Rayleigh length shows that the TFISH signal is indeed showcasing Gaussian divergence. The collinearity of the TFISH with the 800 nm probe as well as its similar Gaussian-like divergence are also strong indicators that the SH signal at the PMT is due to a phase-matched process.

Next, a cylindrical lens is used to replace the probe focusing lens in system case 1 (Fig. 5a). Because the cylindrical focused probe allows more control over the focal intensity, it is used to perform a power-dependence measurement of the TFISH signal. Since the trend was difficult to immediately examine, a log–log plot is shown in Fig. 11b. In accordance with Eq. (1), the power law dictates that the probe energy vs PMT signal should have a slope of 2.0. After background subtraction, our results show a slope of 1.75 which slightly deviates from the expectation. The deviation is indicative of an unknown secondary process producing a strong SH in conjunction with TFISH or unmediated noise.

### Results and discussion: TFISH in single-color air plasma

Using the above experimental setup first without the BBO 1 crystal, the SH due to single color plasma is analyzed. In this subsection, the features of single-color ω plasma pumping are discussed.

#### TFISH polarization dependence and beam spatial pattern

By placing a Glan–Thompson prism with a > 10,000:1 extinction ratio along the propagation of the SH prior to reaching the PMT, the polarization of the SH can be studied. First, the HWPs along the pump and probe arms are both characterized and set onto rotation mounts so that the pump and probe polarizations can be rotated with respect to the fast axis set to the horizontal position.

**Fig. 12:**
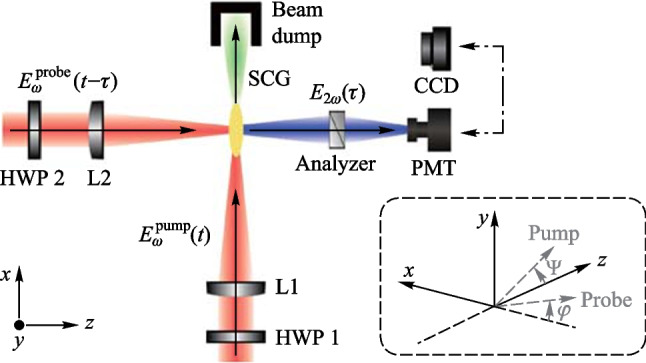
Simplified schematic of Fig. 5 for the purpose of analyzing the polarization in the single-color *ω* pumping scheme. The inset shows in detail the orientation of the polarization of the pump and probe with respect to the Cartesian coordinate defined for the system. SCG: supercontinuum generation. Figure adapted from Ref. [114]

For the polarization experiment, the coordinate axes are redefined with respect to the probe beam as shown in Fig. 12. The probe polarization is characterized by an angle *φ* in the probe beam *x*–*y* plane measured from the *x*-axis. A probe angle *φ* = 0° indicates a p-polarization while an angle *φ* = 90° indicates a probe s-polarization. The optical pump beam polarization has an angle Ψ defined in the probe beam *y*–*z* plane measured from the *z*-axis. For the pump beam, Ψ = 0° and Ψ = 90° represent pump p- and s-polarization, respectively. All measurements are done with a pump energy of 1.2 mJ and probe energy of 300 μJ.

**Fig. 13:**
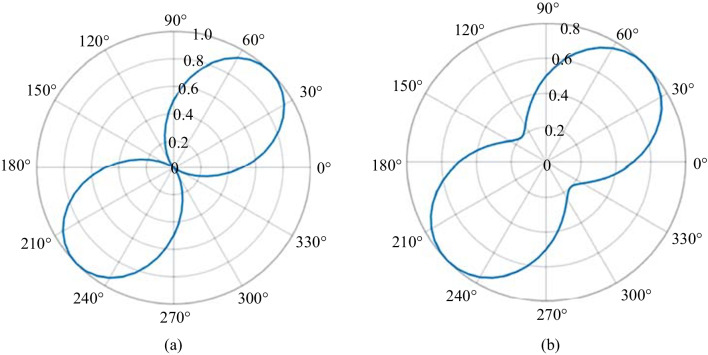
Simulated “Peanut plots” using Stokes vectors for **a** arbitrary light linearly polarized 45° with respect to the optical table and **b** light elliptically polarized with its major axis along the same orientation as that in **a**. The plots are polar representations of Malus’ Law and allow for easy analysis of the polarization state of optical radiation

In the first set of experiments, the pump and probe polarizations are kept to a single value and the Glan prism is rotated to show the polarization distribution of the SH beam. Although no phase information is recovered in these scans, the rotation of the Glan prism can distinguish linear, elliptical, and circular polarizations through “peanut plots” shown in Fig. 13. A simulation of an expected linear state is shown in Fig. 13a, where the angle *θ* = 0° indicates that the Glan prism is aligned to pass p-polarized components. The simulation is made in MATLAB by using a Stokes vector input, passing it through the described system, and visualizing the output as a function of the Glan prism rotation. As such the “peanut plot” shows a 45° linearly polarized beam. In Fig. 13b, an elliptically polarized probe is shown with its major axes aligned along the same axis as the polarization of the linear state in Fig. 13a.

The result is like the linear state, but the orthogonal component to the major axis does not reach zero as predicted by the cosine dependence of the polarization via Malus’ law [84, 123]. Note that because there is no phase information in this system, there is an ambiguity in determining either circularly polarized (in an ambiguous direction), radially polarized, or unpolarized light. All three cases appear as isotropic. To determine if the polarization is circular, a quarter wave plate (QWP) can be used in conjunction with a linear analyzer.

**Fig. 14:**
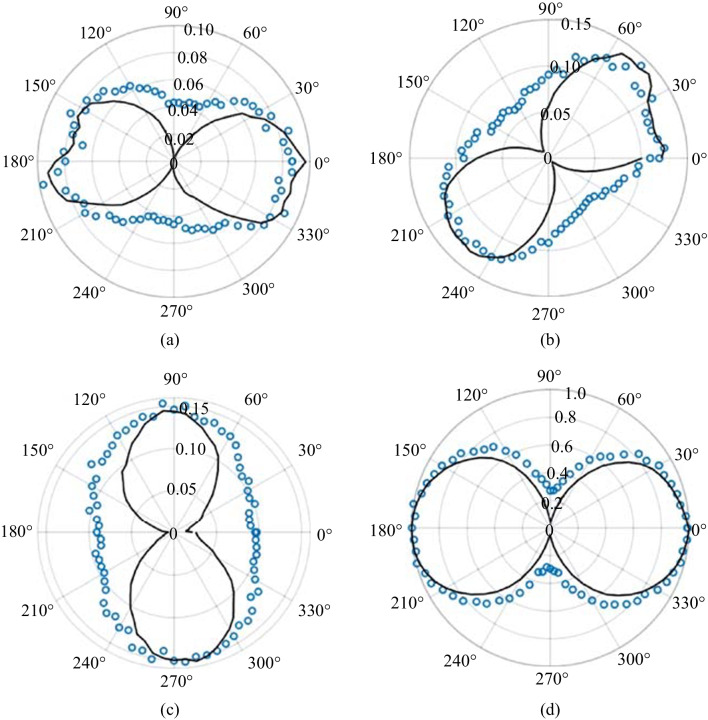
Polarization plots of the TFISH radiation characterized by rotating the Glan analyzer. The polarization is shown for polarization combinations: **a** p–p, **b** p–s, **c** s–p, and **d** s–s. The blue circles are the real data retrieved in the experiment. The black lines represent the data normalized within the range [0 1] for each set such that the overall major axis of the polarization is easy to see. The black lines are representative of the major axis only. The overall magnitudes are normalized to the largest component

The polarization is described in terms of pump-probe polarization configurations (p–p, p–s, s–p, and s–s) in the experiment. Figure 14 shows the polarizations experimentally found as a function of Glan prism rotation. The most notable feature is that the polarization of the SH neither follows the pump or probe polarization. This is important because it remains one of the major reasons to discount SH from the plasma itself as shown in Eq. (2). Rather, the polarization of the TFISH is mostly p-polarized when the pump-probe polarizations are equal (s–s, p–p), and s-polarized when the polarizations are orthogonal (s–p, p–s).

This trend occurs regardless of whether the polarizations are in the same plane (s–s) or along separate planes (p–p). The blue circles are the real data retrieved in the experiment by rotating the Glan analyzer 360° in front of the PMT. The black lines represent the real polarization data normalized within the range [0 1] for each set such that the overall major axis of the polarization is easy to see. The strongest SH signal is found when the s–s configuration is used, and all other configurations can only reach 15% of the signal that the s–s case provides. This is because for all other polarization combinations, the probe undergoes a projection:

3 $$\rho \left({\theta }_{\mathrm{C}}\right)= \left[\begin{array}{ccc}\mathrm{cos}\,{\theta }_{\mathrm{C}}& 0& \mathrm{sin}\,{\theta }_{\mathrm{C}}\\ 0& 1& 0\\ -\mathrm{sin}\,{\theta }_{\mathrm{C}}& 0& \mathrm{cos}\,{\theta }_{\mathrm{C}}\end{array}\right],$$

where *θ*_C_ is the noncollinear angle (90° in this system). When analyzing the interaction between the Jones or Stokes vectors ${\left|\left[{\overrightarrow{S}}_{\mathrm{pump}} \cdot {\overrightarrow{S}}_{\mathrm{probe}}\right]\right|}^{2}$, the s-components as a function of angle of noncollinearity remain unchanged while the projection of the p-components causes the signal to fall as a function of ${\mathrm{cos}}^{2}{\theta }_{\mathrm{C}}$.

**Fig. 15:**
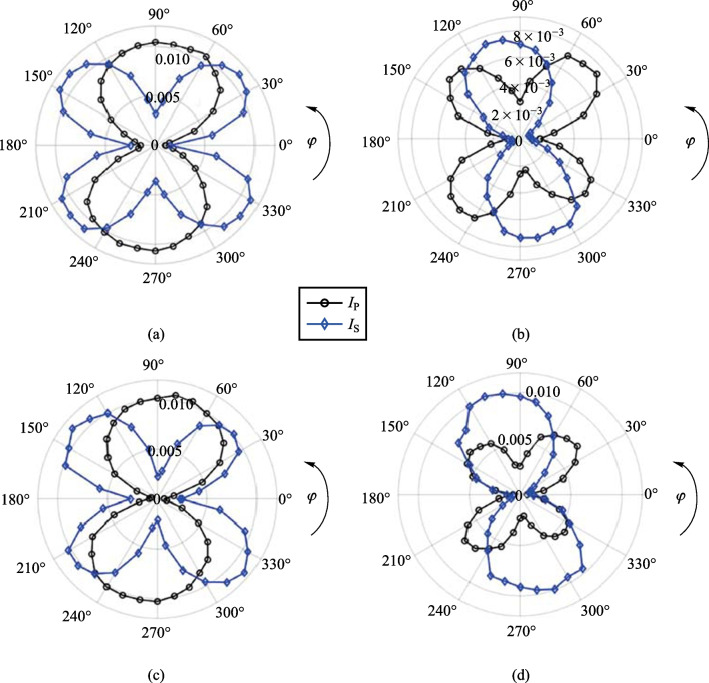
Experimental data plots of the s-component (blue diamonds) and the p-component (black circles) of the TFISH 2*ω* as the probe beam polarization is rotated from *φ* = 0° to *φ* = 360° and the pump beam polarization is kept static at **a** Ψ = 90°, **b** Ψ = 165°, **c** Ψ = 275°, and **d** Ψ = 335°

The possibility of plasma birefringence quickly comes to mind in explaining the rotation of the polarization [124–127]; however, the short interaction region and alignment of the polarizations with respect to density gradients discount this notion. To further investigate the origin of the polarization changes, either the HWP along the pump or probe are rotated such that *φ* or Ψ undergo a rotation from 0° to 360° while the other HWP remains static at either 0 or 90°. When analyzing the p- or s-component alone (setting the Glan analyzer to 0° or 90°), an asymmetric dependence on the input rotated pump or probe polarization is shown in Fig. 15. In the case where Ψ = 90° and *φ* rotates, the p-component of the SH shows a twofold mirror symmetry while the s-component shows a fourfold symmetry with respect to input probe polarization as shown in Fig. 15a. Moreover, when Ψ is rotated as well, the twofold and fourfold symmetry seem to oscillate as shown in Fig. 15b–d.

The component symmetries explain why the s–s and p–p configurations lead to p-polarized SH while orthogonal configurations lead to s-polarized SH. In Fig. 15a, when *φ* = 90°, the p-component of the SH is larger in magnitude than the s-component. However, when *φ* = 0° or 360°, the s-component dominates. The ratio of the two components determines the magnitude of the signal [114]. For this reason, in Fig. 14, the s–s case is dominant and mostly leads to p-polarized SH. The s-p case has components in s that are only slightly larger than the p components. This leads to a nearly circular state with very low magnitude.

**Fig. 16:**
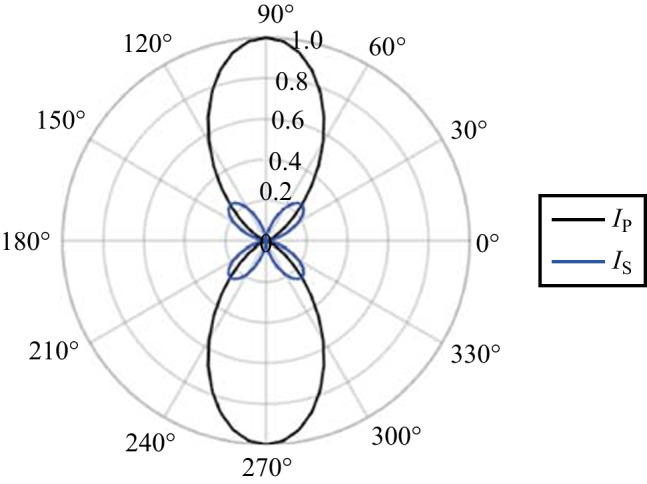
Simulated plot of the s- and p-component of the polarization of a linear mode after passing through a rotating linear polarizer for initial input p-polarized stokes vector and a rotating analyzer set to pass s-polarized light

Aside from the changes in magnitude of the SH s-polarized component with respect to the p-polarized component, the observation is reminiscent of the plasma acting as a rotating linear polarizer. A simulation of a rotating linear polarizer in the interaction region using Stokes vectors is shown in Fig. 16 to agree well with this description. An input p-polarized state is made incident on a rotating analyzer set to pass s-polarized light at *φ* = 90°. Evaluation of the s- and p-components shows the same twofold and fourfold symmetry as seen in the experiment. The rotating linear polarizer interpretation is a manifestation of a radially polarized electric field being involved in the SH process. It is, however, important to note that the same asymmetry is noticed in *χ*^(2)^ materials when generating SH [30, 128–131]. Under the latter interpretation, the plasma would act as a C_4ν_ point group material. However, the symmetry conditions for materials in this point group do not apply to the air plasma, the allowed tensor elements do not match the experimentally recovered polarizations, and Eq. (2) appears to be violated.

**Fig. 17:**
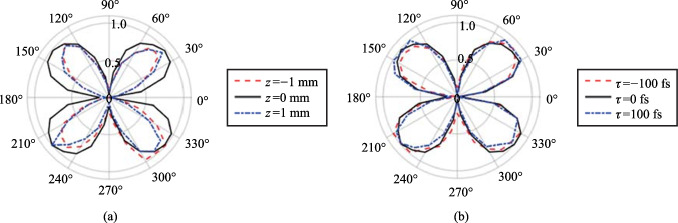
Polarization asymmetry dependence observation of the TFISH s-polarized component as the probe is rotated for the case of Ψ = 90° along **a** different P-scan positions, and **b** different temporal pump-probe delay times. The signals are normalized to their respective maximum values

Plasma grating structure formation has been used before to show that a laser-induced plasma could act as either a waveplate or linear polarizer [132, 133]; however, in those studies, the presence of an SH field was not noted, and the changes occurred on the probe beam. Interestingly, in Refs. [132, 133], the polarizer effect depends crucially on a secondary beat wave (such as an ionization field). This means that as the P-scan position is changed—and therefore the localized electron density is changed—the effectiveness of the linear polarizer is varied. However, as seen in Fig. 17, when the symmetry plots are gathered at different z positions of the P-scan trace or at different correlation times *τ*, the overall pattern remains the same. Since only the magnitude of the s-component changes, and because the p-component is coupled to the s-component as seen in Fig. 15, the polarization of the SH remains unchanged along the P-scan and at different times.

An alternative explanation for the symmetry plots can be found by considering the TFISH expression in Eq. (1). If the THz electric field is along the same polarization as the pump, when the field *E*_THz_ is matched to the probe polarization, the interaction is maximized. Likewise, when *E*_THz_ is orthogonal to the probe, the interaction is minimized. The effect is like a linear polarizer with its orientation along the pump polarization.

**Fig. 18:**
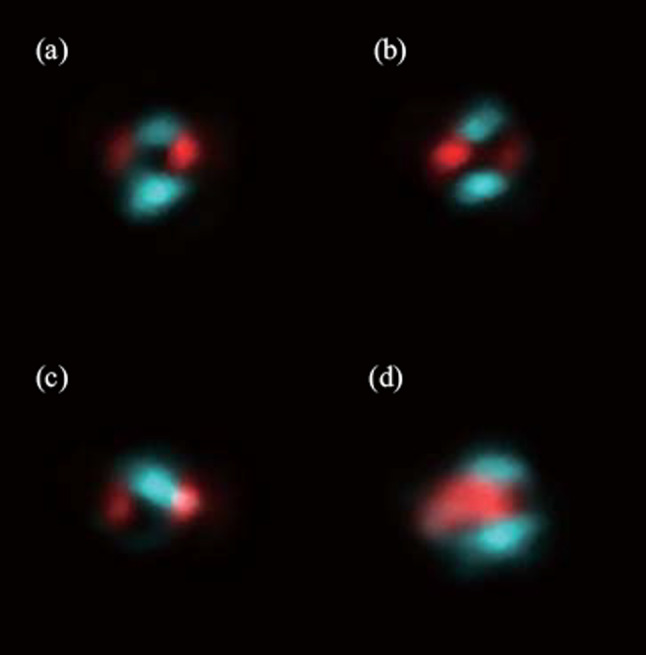
Showcase of radially polarized TFISH beams as gathered by a CCD and an analyzer. The images are false color plots where the red color indicates the detected p-component of the SH, and the blue indicates the detected s-component of the SH. The plots are shown for pump-probe polarization configurations: **a** p–p, **b** p–s, **c** s–p, and **d** s–s

Next, the PMT is replaced with a CCD to examine the effect of the polarization on the beam pattern of the TFISH radiation. In Fig. 18, the pump is kept at either p (a and b) or s (c and d) polarization. The analyzer is used to pass either only the p- or s-component of the SH. The images are false color plots where the red color indicates the detected p-component of the SH and the blue indicates the detected s-component of the SH. Figure 18a shows the beam pattern for a p–p configuration, (b) shows the pattern for p–s configuration, (c) shows the pattern for an s–p configuration, and (d) shows the pattern for an s–s configuration. Contrary to the expression in Eq. (2), the pattern appears conical and the polarization itself appears to describe a radially polarized SH. This result is encouraging as the TFISH/EFISH process is expected to be radially polarized assuming a radially polarized THz or charge separation-induced electric field [57].

**Fig. 19:**
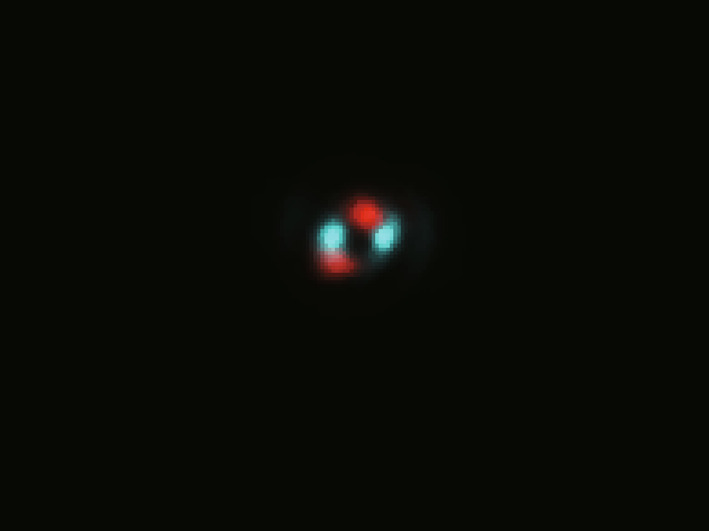
Image of nearly azimuthally polarized SH beam pattern gathered with a CCD and an analyzer. The beam pattern is shown for a p-pump and 45° probe beam

Interestingly, the polarization of the SH can be switched to near azimuthal by rotating the probe to 45° polarization in the p-pump case. As seen in Fig. 19, the SH pattern remains conical with an azimuthal polarization description. The mechanism for this switching is not known at this time, but the birefringence of the plasma may play a key role in this case.

#### THz ABCD

Because the above experiments have largely only left TFISH and EFISH as viable sources of the SH observed, and because TFISH and EFISH are mathematically equivalent except for the nature of the electric field in question, a TFISH model is used in this section to explain the SH observed. The single-color coherent radiation is evaluated by removing the pump BBO and increasing the probe intensity near 1 W to produce a secondary plasma. In this experiment, the probe beam plasma produces a local electric field that acts as a local oscillator termed here as an ionization-induced second harmonic (IISH) signal. Given that the probe beam has a frequency *ω* and the THz has a frequency ${\omega }_{\mathrm{THz}} \ll \omega$, if the material is exposed to sufficiently large intensity, a process ${\chi }^{\left(3\right)}\left(2\omega = \omega + \omega \pm {\omega }_{\mathrm{THz}}\right)$ can be enacted. The interference mixing of the TFISH signal with the IISH signal has the form [37]:

4 $${S}_{2\omega } \propto {\int }_{-\infty }^{\infty }{\left|{\chi }_{ijkl}^{\left(3\right)}\right|}^{2}{\left|{E}_{\omega }\left(t - \tau \right)\right|}^{4}{\left|{E}_{\mathrm{THz}}\left(t\right)\right|}^{2}\partial t+2{\int }_{-\infty }^{\infty }\mathfrak{R}\left\{{\left|{\chi }_{ijkl}^{\left(3\right)}\right|}^{2}{\left|{E}_{\omega }\left(t - \tau \right)\right|}^{4}{E}_{\mathrm{THz}}\left(t\right){E}_{\mathrm{Pl}}^{*}\left(t\right)\right\}\partial t + {\int }_{-\infty }^{\infty }{\left|{\chi }_{ijkl}^{\left(3\right)}\right|}^{2}{\left|{E}_{\omega }\left(t - \tau \right)\right|}^{4}{\left|{E}_{\mathrm{Pl}}\left(t\right)\right|}^{2}\partial t,$$

where *S*_2*ω*_ is the detected PMT signal, ${\chi }_{ijkl}^{\left(3\right)}\left(2\omega ,\omega ,\omega ,{\omega }_{\mathrm{THz}}\right)$ is the third-order susceptibility tensor of air within the plasma, ${E}_{\omega }\left(t \pm \tau \right)$ is the electric field of the probe beam delayed by a temporal factor *τ*, ${E}_{\mathrm{THz}}\left(t\right)$ is the THz field confined to the plasma filament, and ${E}_{\mathrm{Pl}}\left(t\right)$ denotes the plasma electric field corresponding to the nonlinear current induced in the plasma filament by the probe beam. The susceptibility subscripts *i*,* j*, *k*, and *l*, respectively indicate the polarization of the SH, two probe fundamental (for both *j* and *k*), and THz fields. Finally, $\mathfrak{R}$ is the real part of the expression.

**Fig. 20:**
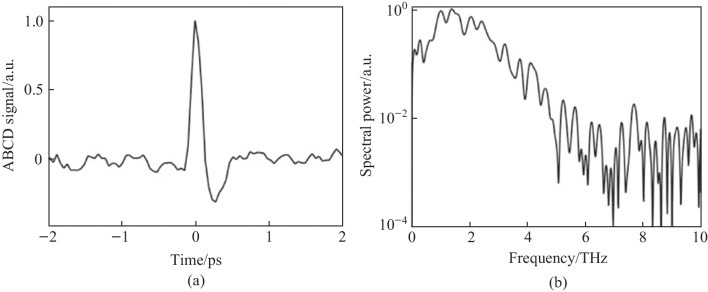
**a** Temporal ABCD signal. **b** Spectral power gathered from the Fourier transform of **a**. Figures adapted from Ref. [113]

The first term resulting from Eq. (4) is the “incoherent” TFISH signal as evaluated in Sect. 2.2.1 and Fig. 6. The second term in Eq. (4) is the air-breakdown coherent detection (ABCD) term [37]. Furthermore, the plasma field itself induces a THz field as well. This means that even if the IISH term is isolated from the TFISH term, a second TFISH term may be formed. In the case of a weak ionization regime, the second harmonic signal is dominated by the TFISH and coherent term nearly equally. If the optical intensity is increased beyond a certain regime, the plasma term will begin to saturate and since it is coupled to the coherent term, the coherent term eventually becomes dominant over the TFISH term. Unfortunately, the instability of the plasma term severely limits the dynamic range and signal to noise of this measurement method. The third term in Eq. (4) can be removed through the lock-in amplifier detection and Fig. 20 shows the temporal ABCD signal measured by PMT and its corresponding spectrum.

Unfortunately, the second term is presented as a spectral filtering operation where the frequency spectra of the THz is filtered by the probe beam Gaussian spectrum—this can be seen via the area property of the Fourier transform. What this means is that the detectable spectrum is now filtered through ${\int }_{-\infty }^{\infty }{I}_{\omega }^{2}\left(t - \tau \right)\partial t$ by a factor $\sqrt{2}/{\tau }_{\mathrm{p}}$.

### Results and discussion: TFISH in two-color air plasma

In the next subsection, the two-color pumped TFISH experiments are detailed. Figure 5 provides a sketch of the system and the two cases evaluated in this section: case 1 where the two-color source is produced by placing BBO 1 in front of lens 1, and case 2 where a Mach–Zehnder type phase compensator is used to control the phase between the pump *ω* and 2*ω* frequencies.

#### TFISH study of plasma dynamics

The experiments presented in this subsection use case 1 in Fig. 5a. The very high conversion efficiency (> 0.01%) in the two-color pumped system allows for very sensitive study of the THz source and plasma dynamics. The physical dynamics of the formation of THz within a plasma filament have been previously explored experimentally, but the methods used require materials that will be damaged by the created plasma. In addition, the indirect detection methods used may be prone to imaging errors through the THz optics. Considering that the system P-scan yields information about the confinement of THz along the length of the plasma filament as suggested in Ref. [134], a non-contact TFISH probe for the THz was developed.

**Fig. 21:**
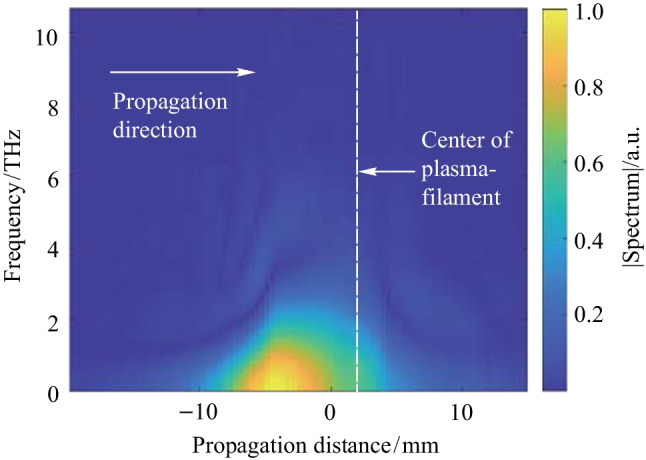
2D view of the spectral buildup of the THz frequency along the plasma length measured by analyzing the TFISH spectrum along every point of a plasma scan trace. The zero position indicates the position of the strongest TFISH, the dashed line shows the estimated center of the plasma filament by visual inspection, and the arrow shows the laser propagation direction. Figure taken from Ref. [113]

Moving along the plasma length at an interval of 0.5 mm, the TFISH and its corresponding spectrum for every point is retrieved and plotted onto Fig. 21. This figure then can be interpreted as the build-up of the THz spectrum as a function of propagation along the plasma filament. The spectrum is low in areas of perceived lower electron number density owing to the nonlinear current along the plasma filament having oscillations in the low-frequency THz region. Viewed in conjunction with the P-scan trace in Fig. 8b, the TFISH signals retrieved at the tail of the plasma at propagation distance *z* =  − 10 mm are temporally broad and lead to narrow TFISH spectra. After the maximum spectrum is reached beyond *z* = 0 mm, the THz begins to diverge and leave the filament. This is seen by a decrease in the spectral intensity but comparatively minimal loss in the bandwidth of the TFISH.

**Fig. 22:**
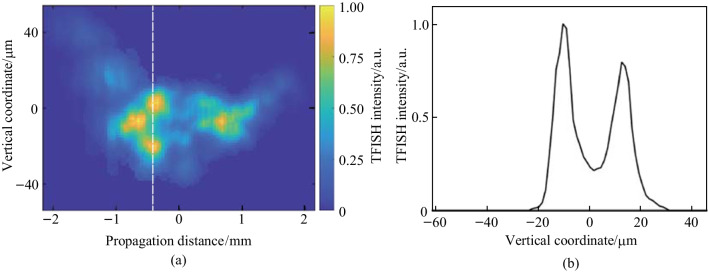
**a** Plasma transverse profile reconstruction gathered by stitching cylindrical TFISH slices along various plasma scan points. **b** Transverse THz profile gathered from the cylindrical focusing on a single plasma scan point in **a**. The dash line shows the location of the transverse line cut. Figures taken from Ref. [113]

By changing lens 2 to a 50 mm cylindrical lens and using case 2 in Fig. 5b, vertical slices can be captured along the plasma with the noncollinear TFISH system. The PMT is replaced with a conventional CCD (Imaging Source DMK 27BUP031) and the cylindrical focus is directly imaged with the 4F imager. Cylindrical focusing allows for a TFISH signal to be spatially correlated with the THz field along the plasma in the vertical direction so that the spatial vertical profile of the THz can be shown. The initial alignment of the system is done by removing the bandpass filters to observe the residual 800 nm probe beam. At the focus, a 1 dimensional (1D) Airy function is found due to the strong cylindrical focusing. The filters are then placed to confirm the 1D cylindrical focus Airy function for the TFISH signal. The result is then a plasma slice with a vertical extent of 50 μm denoting the thickness of the plasma induced by the pump beam. The horizontal width of the slice then shows the width of the TFISH beam spot measured as 1/e^2^ (15 μm). A vertical TFISH slice is recorded every 43 µm along a plasma scan trace to produce Fig. 22a. Note that since the case 2 pump lens has a shorter focal length, the induced plasma is much smaller compared to the plasma scan shown in Fig. 8b. The temporal matching between the pump and probe is maintained so that the evolution of the THz signal can be followed through the plasma scan. Although the full extent of the plasma cannot be recorded due to the decreased sensitivity of our CCD along the extreme ends of the plasma, Fig. 22a shows (coupled along with the findings in Fig. 21) that as the THz spectrum grows in the plasma, the transverse profile of the radiation becomes conical in nature. At the zero position, TFISH intensity is lowered likely due to subsequent absorption of the THz in high electron density regions of the plasma. Beyond zero, the pattern reemerges. This serves as a visual representation of the filament refocusing after plasma absorption and defocus. A transverse line-cut of the conical emission is shown in Fig. 22b. The expected double-lobe Gaussian is seen and the result matches previous experimental findings in Ref. [135].

#### TFISH polarization dependence and double-pump behavior

Here, the polarization dependence of the two-color pumped source is discussed in detail. The polarization measurements are carried out in case 2 in Fig. 5b because the Mach–Zehnder platform allows for the control of the three polarizations (pump *ω*, pump 2*ω*, and probe *ω*) independently as well as the timing between all three polarized waves. The same Glan prism analyzer used in Sect. 2.3.1 is used to determine the polarization of the SH.

**Fig. 23:**
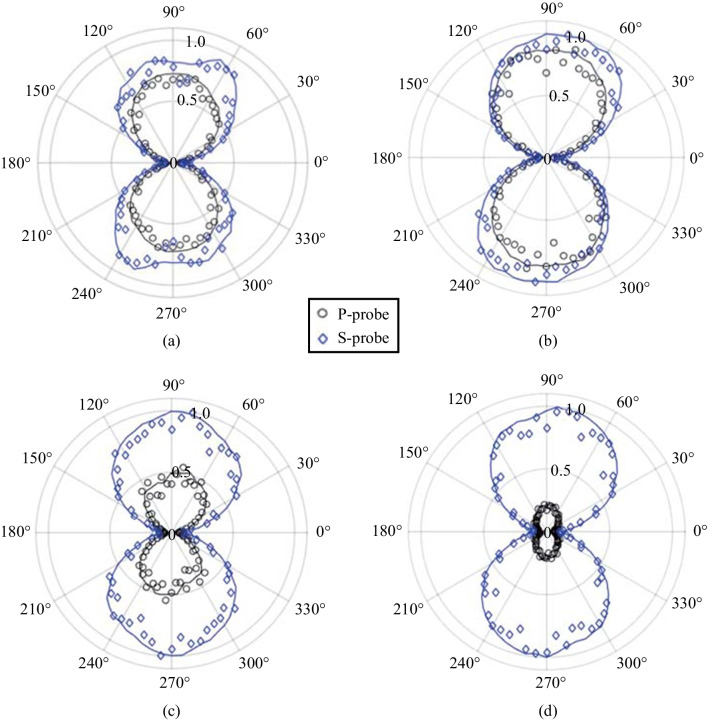
Polarization plots of the two-color pumped TFISH radiation characterized by rotating the Glan analyzer. The polarization is shown for polarization combinations (*ω* pump-2*ω* pump): **a** p–p, **b** p–s, **c** s–p, and **d** s–s. The blue diamonds are the real data retrieved in the experiment for s-polarized probe beam while the black circles are real data retrieved for p-polarized probe beam. The blue and black lines are smoothened data to show the overall trend for s and p-polarized probe, respectively. The plots are normalized to the largest signal value

For the polarization analysis, the permutation convention *ω*-pump 2*ω*-pump is used. Figure 23 shows the polar plot characterization for all polarization permutations. Surprisingly, the polarization resulting from the two-color operation is nearly always a linear s-polarized state regardless of the input permutation values. The p-probe is noticeably weaker than the s-probe case in all permutations due to the geometrical factor (the noncollinear angle). To be able to understand the reason for the polarization dependence, the temporal dynamics between the pump polarizations must be gathered. By delaying the 2*ω* path by a considerable amount, the noncollinear TFISH system showed a behavior like the double-pump phenomena in THz experiments [115–120].

**Fig. 24:**
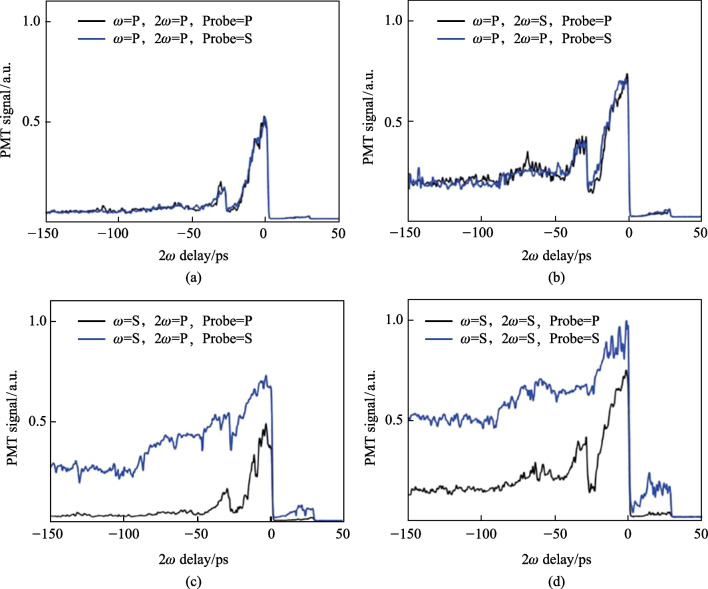
Double-pump phenomena retrieved by delaying the pump 2*ω* while keeping the pump ω static in time. The blue lines and black lines represent the use of an s- or p-polarized probe, respectively. The four cases shown are for the pump *ω*—pump 2*ω* permutations: **a** p–p, **b** p–s, **c** s–p, and **d** s–s. No Glan analyzer is used. The signals are normalized to the largest signal

The 2*ω* delay is controlled by the delay stage along the 2*ω* path in case 2 (Fig. 5b). The main delay stage (the stage that controls the timing between the pump plasma and the probe) is set to the peak of the TFISH signal. In Fig. 24, when the delay is negative, the 2*ω* pump field arrives at the same spatial location as the *ω* pump, but much later in time. Because of this, the noncollinear TFISH system can only read the TFISH signal from the *ω* pump. As the delay approaches zero, the *ω* beam plasma acts as a pre-pulse plasma and has its TFISH signal enhanced by the arrival of the 2*ω* plasma. At positive delays, the signal sharply drops to the system noise level. Here, the 2*ω* pump arrives earlier than the* ω* pump temporally. In the latter case, the 2*ω* plasma serves to absorb the THz signal and therefore the TFISH signal sharply decreases. Conversely, in the case that the *ω* pump arrives to the focus first and creates its plasma, the enhancement seen is indicative of the rectification enhancement that the 2*ω* has on the* ω* beam. Although the two fields have short pulse duration of about 100 fs and the coherence between them is small, the long temporal dynamic in Fig. 24 can be understood as consequence of the fact that the plasma induced by the two separate fields lasts longer that the individual pulses temporally. As such, the 2*ω* pump plasma can continue to interact with the temporally static *ω* pump.

Since the TFISH polarization does not follow the pump or probe polarization explicitly (especially in the p-p and s–s permutation), the expression in Eq. (2) is violated. This is further proof that a *χ*^(2)^ process is minimally involved in the production of SH. Additionally, the TFISH polarization is notably more linear compared to all the single-color cases evaluated in Sect. 2.3.1. When the pump *ω* is p-polarized, the TFISH signal is minimized and the 2*ω* pump delay shows that the temporal dynamics of the double-pumping are minimized. For all four permutations, the TFISH signal appears to be optimized when the ω pump component is s-polarized and an s-polarized probe is used. The polarization of the probe must also match the pump *ω* polarization to yield the strongest signal in all permutations. As such, it can be surmised that the THz field (or charge separation-induced *E*-field) follows the general polarization of the *ω* pump. This is an encouraging result matching previous work in two-color air plasma systems [112].

Using a polarization model ${\chi }_{ijkl}^{(3)}(2\omega = \omega + \omega \pm {\omega }_{\mathrm{THz}})$, the TFISH polarization can be explained. In the p-polarized *ω* pumped system, the expected polarization according to the FWM model is a p-polarized TFISH signal [136] since a p-polarized THz is expected. However, because the noncollinear angle limits the system capability for p-polarized components, no significant p-polarized SH is noted. Instead, the plasma asymmetry allows for the components ${\chi }_{yxxx}^{(3)}$ or ${\chi }_{yyyx}^{(3)}$ to have a nonzero value as depicted in Figs. 23a and 24a. The symmetry breaking effect has been noticed before in Ref. [112]. For the p-s permutation, the angle between the pump *ω* and pump 2*ω* is 90°. According to Ref. [136], an s-polarized THz field would be expected. Two components of the susceptibility tensor are possible: ${\chi }_{yxxy}^{(3)}$ and ${\chi }_{yyyy}^{(3)}$. The ${\chi }_{yyyy}^{(3)}$ configuration should be stronger than the ${\chi }_{yxxy}^{(3)}$ case, but due to the geometrical factor and the mismatch between the two pump polarizations, the signals found are of similar order as seen in Figs. 23b and 24b. For the s-p permutation, the ${\chi }_{yyyy}^{(3)}$ component is the strongest, but its magnitude will be reduced because the THz field magnitude does not reach its maximum value—since the angle between the pump *ω* and pump 2*ω* polarizations is 90° in Figs. 23c and 24c. This explains why the s-probe case is stronger than the p-probe. Lastly, in the s–s permutation, the susceptibility tensor is given by ${\chi }_{yyyy}^{(3)}$, and ${\chi }_{yxxy}^{(3)}+{\chi }_{yxxx}^{(3)}$. Because both pump polarizations are matched, Figs. 23d and 24d show the maximum value for the TFISH signal. Unlike the single-color case, the two-color case shows linear values for the SH which in turn imply linear THz polarization.

#### THz OBCD

To retrieve a coherent signal for the two-color field, a secondary 100 μm BBO crystal (BBO 2) is used to generate an SH field to act as a local oscillator as shown in Fig. 5b. The BBO 2 crystal is placed along the probe beam path and rotated so that the SH polarization matches the polarization of the TFISH signal in the plasma. The interference is then imaged by the 4F imaging system onto the PMT. The coherent signal resulting from this method, known as an optically biased coherent detection (OBCD), is theoretically described by a process [67]:

5 $${S}_{2\omega } \propto {\int }_{-\infty }^{\infty }{\left|{\chi }_{ijkl}^{\left(3\right)}\right|}^{2}{\left|{E}_{\omega }\left(t - \tau \right)\right|}^{4}{\left|{E}_{\mathrm{THz}}\left(t\right)\right|}^{2}\partial t+2{\int }_{-\infty }^{\infty }\mathfrak{R}\left\{{\chi }_{ijkl}^{\left(3,\text{*}\right)}{\chi }_{ijk}^{\left(2\right)}{\left|{E}_{\omega }\left(t - \tau \right)\right|}^{4}{E}_{\mathrm{THz}}\left(t\right)\right\}\partial t+ {\int }_{-\infty }^{\infty }{\left|{\chi }_{ijk}^{\left(2\right)}\right|}^{2}{\left|{E}_{\omega }\left(t - \tau \right)\right|}^{4}\partial t.$$

**Fig. 25:**
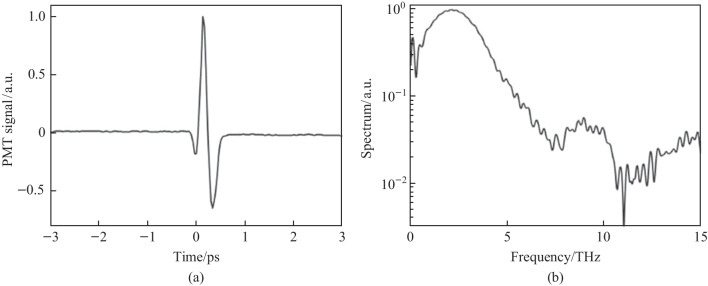
**a** Temporal OBCD signal. **b** Spectral power gathered from the Fourier transform of **a**. The signals were obtained at a *ω* optical pulse duration near 110 fs as measured with an intensity autocorrelator. Figures adapted from Ref. [113]

In Eq. (5), ${\upchi }_{ijk}^{\left(2\right)}$ denotes the BBO second-order susceptibility. A secondary probe wave ${E}_{2\omega } = {\upchi }^{\left(2\right)}\left(2\omega = \omega + \omega \right){E}_{\omega }^{2}\left(t -\uptau \right)$ is generated. Equation (5) is like the signal term as shown in Eq. (4). The second term in Eq. (5) is akin to the ABCD term as shown in Ref. [37]. This term is denoted as the “coherent” signal as it is directly proportional to the THz electric field rather than the intensity. The third term can be mostly removed through the lock-in and denotes the BBO-induced LFISH. In Fig. 25, the OBCD signal is gathered along with its spectrum—showcasing a broad bandwidth of detection matching the conventional ABCD methods.

Since the pump plasma fluctuations also modulate the probe beam, a lock-in amplifier used to detect the THz signal cannot fully eliminate the background in the temporal trace. Therefore, some DC components appear in the signal spectrum if the magnitude of the local oscillator signal is too high. Unlike the ABCD-II method, because the LFISH comes directly from the probe beam, the TFISH term also does not vanish with a scan (the BBO LFISH is tied directly to the TFISH). Because of this, the magnitude of the BBO-induced SH becomes important—If it is weaker than the TFISH term, the TFISH signal will dominate over the coherent signal. Likewise, if the BBO-induced SH is too strong, issues can occur in detecting photoelectrons at the PMT.

The OBCD system also brings new complications in that the polarizations of the TFISH and LFISH need to be matched to yield the best system. This is difficult because a crystal such as BBO will produce SH at its highest yield along the extraordinary axis when the input *ω* waves are incident with polarization along its ordinary axis [30]. This means that the crystal azimuth and rotation angles are essential in determining the tradeoff between the proper polarization projection and the conversion efficiency of the probe. However, the OBCD brings an advantage in planning for future experiments such as single-shot detection of THz waves. The SH signal produced by OBCD will always be greater than that of either ABCD signal while using weak probe beams since ${\upchi }_{ijk}^{\left(2\right)} \gg {\upchi }_{ijkl}^{\left(3\right)}{E}_\mathrm{{Pl,b}}\left(t\right)$ for most usable materials [30]. This is especially true for materials with larger *χ*^(3)^ as was shown in Ref. [39].

Next, a Golay cell is used to characterize the far-field THz radiation simultaneously. As shown in Fig. 5, the Golay cell is placed along the pump beam path at a 2F configuration from the laser-induced plasma. The supercontinuum is blocked due to: (1) the high-density polyethylene (HDPE) material of the THz lens used, (2) a Si wafer placed along the diverging portion of the far-field THz, and (3) a combination of low pass filters and a second Si wafer at the Golay cell window. The system is optimized to maximize the Golay cell signal and a correlation to the TFISH signal is noticed.

**Fig. 26:**
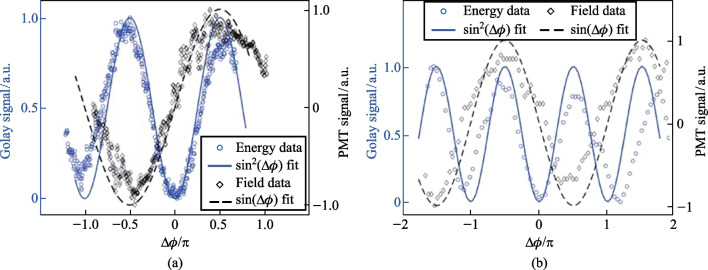
**a** Phase scan measured by moving the BBO 1 toward the plasma in case 1. **b** Phase scan measured by scanning the wedges in case 2. The blue circles and black diamonds represent the data of the energy and field detection, respectively. The blue solid line and black dashed lines represent the ideal fit for the THz energy and field dependence as the phase between *ω* and 2*ω* is varied. Figures taken from Ref. [113]

By translating BBO 1 along the* z* axis between the lens and the focal point of case 1 (Fig. 5a), the phase between the pump *ω* and 2*ω* is changed according to the expression:

6 $$\Delta\varphi= \frac{2\uppi {\left[n\left(2\omega \right) - n\left(\omega \right)\right]}_{\mathrm{air}}L}{{\lambda }_{2\omega }},$$

where *n* indicates the index of refraction, *L* is the BBO to focus distance and *λ* denotes the wavelength. As *L* is changing, the Golay cell is used to evaluate the change in THz energy. As expected, Fig. 26a shows that the THz energy has a sin^2^(∆$\varphi )$ dependence to *L* while the OBCD signal has a sin(∆$\varphi )$ dependence attributed to the dephasing between both optical frequencies in the plasma filament [112]. The plasma scan (Fig. 8b) trace in conjunction with the phase delay scan (Fig. 26) can be used to deduce an estimate for the electron number density in the plasma filament. For this procedure, the phase shift induced inside a plasma [137], ${\upphi }_{\mathrm{plas}} = {k}_{\mathrm{o}}{n}_{\mathrm{plas}}L = \frac{2\uppi L}{{\lambda }_{\omega }}\left(1 - \frac{{N}_{\mathrm{e}}}{2{N}_{\mathrm{c}}}\right)$ is used, where *N*_e_ is the electron number density and *N*_c_ is the critical density. To find the phase difference in the filament, $\Delta{\upphi }_{\mathrm{fil}} = {\upphi }_{\mathrm{plas}} - {\upphi }_{\mathrm{air}} = \frac{2\uppi L}{{\lambda }_{\omega }}\left(\frac{{N}_{\mathrm{e}}}{2{N}_{\mathrm{c}}}\right)$. Comparing this to the phase shift induced by shifting the BBO position (Eq. (6)) and solving for the electron density gives the expression:

7 $${N}_{\mathrm{e}} = \left(\frac{{\lambda }_{2\omega }}{L}\right){N}_{\mathrm{c }}.$$

In case 2 (Fig. 5b), the same experiment can be conducted by shifting the path between *ω* and 2*ω* with a set of wedges. In this case, the phase is changed according to the expression:

8 $$\Delta\varphi= \frac{2\uppi {\left[n\left(2\omega \right) - n\left(\omega \right)\right]}_{\mathrm{W}}\Delta{z}\text{ tan}{\theta }_{\mathrm{W}}}{{\lambda }_{2\omega }},$$

where the W subscript indicates a property of the wedge, Δ*z* is the relative positioning of the wedges, and *θ* is the wedge angle. Using the Golay cell as before, Fig. 26b showcases that the shift in the far-field energy THz corresponds well with a shift in the OBCD signal. Using the formalism of Eq. (8), the electron density can also be estimated by the expression:

9 $${N}_{\mathrm{e}} = \left(\frac{{\lambda }_{2\omega }{\left[n\left(2\omega \right) - n\left(\omega \right)\right]}_{\mathrm{air}}}{{\Delta z}{\left[n\left(2\omega \right) - n\left(\omega \right)\right]}_{\mathrm{W}}\mathrm{tan}{\theta }_{\mathrm{W}}}\right){N}_{\mathrm{c}}.$$

Using either Eq. (7) or Eq. (9) in conjunction with Fig. 26, the number density is 3 × 10^16^ cm^−3^, which matches the accepted literature values for similar experiments [109, 110]. This experiment shows that the perceived OBCD signal is indeed related to the nonlinear rectified current on the plasma filament as theorized in Ref. [34]. Moreover, the electron density also sets the characteristic *T*_p_ on the order of > 500 fs which is significantly longer than the laser optical pulse duration. This means that the EFISH/TFISH process should be dominated by the TFISH effects as discussed in Sect. 2.1.2. For a more localized measurement of the electron density, the phase compensation can be included in the probe beam as well. Since the OBCD is a phase-sensitive measurement method, the local changes can be obtained for every P-scan point by performing the phase delay on the probe rather than the pump.

### System metrics

Next, the system dynamic range and signal to noise ratio were gathered to quantitatively compare the system to existing THz detection modalities. The mathematical definition of the signal to noise ratio (SNR) is given by the ratio of the system signal to the signal noise fluctuation. This is represented by the quantity:

10 $$\mathrm{SNR }= \frac{{S}_{2\omega }}{{N}_{2\omega }},$$

where ${S}_{2\omega }$ is the OBCD signal as previously discussed and ${N}_{2\omega }$ is the system signal noise fluctuation. By extending the formulation in Ref. [69], the SNR for an OBCD system is given by the expression:

11 $${\mathrm{SNR}}_{2\omega } \propto \frac{{\int }_{-\infty }^{\infty }\mathfrak{R}\left\{{\chi }_{ijkl}^{\left(3,\text{*}\right)}{\chi }_{ijk}^{\left(2\right)}{I}_{\omega }^{2}\left(t - \tau \right){E}_{\mathrm{THz}}\left(t\right)\right\}\partial t}{{\int }_{-\infty }^{\infty }{I}_{\omega }\left(t - \tau \right)\Delta {I}_{\omega }\left[{\left|{\chi }_{ijkl}^{\left(3\right)}\right|}^{2}{I}_{\mathrm{THz}}\left(t\right) + 2\mathfrak{R}\left\{{\chi }_{ijkl}^{\left(3,\text{*}\right)}{\chi }_{ijk}^{\left(2\right)}{E}_{\mathrm{THz}}\left(t\right)\right\} + {\left|{\chi }_{ijk}^{\left(2\right)}\right|}^{2}\right]\partial t}.$$

Above, $\Delta {I}_{\omega }$ is taken as the laser intensity fluctuation. Similarly, the dynamic range (DR) is gathered as the ratio of the signal to the background noise (where the THz electric field is equal to zero). This is represented by the quantity:

12 $${DR}_{2\omega } \propto \frac{{\int }_{-\infty }^{\infty }\mathfrak{R}\left\{{\chi }_{ijkl}^{\left(3,\text{*}\right)}{\chi }_{ijk}^{\left(2\right)}{I}_{\omega }^{2}\left(t - \tau \right){E}_{\mathrm{THz}}\left(t\right)\right\}\partial t}{{\int }_{-\infty }^{\infty }{\left|{\chi }_{ijk}^{\left(2\right)}\right|}^{2}{I}_{\omega }\left(t - \tau \right)\Delta {I}_{\omega } \partial t}.$$

Experimentally, the SNR is found as the ratio of the peak of the OBCD signal to the standard deviation of the signal. The standard deviation is found by recording data over a given period while locked to the peak of the OBCD signal. The DR is similarly found as the ratio of the peak OBCD signal to the signal retrieved at long time delays where the pump and probe no longer have interaction.

**Fig. 27:**
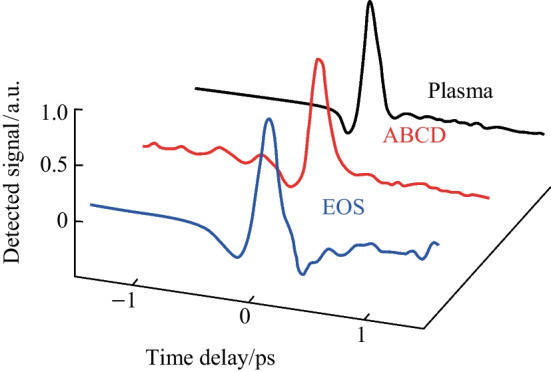
Plot compiling the temporal waveforms gathered by a conventional ABCD, EOS, and plasma OBCD system used to analyze the same plasma source. The spectrum can be computed by performing a Fourier transform on these waveforms

A conventional two-color ABCD system as shown in Fig. 3 was constructed with the same plasma source described in the previous sections by collecting the far-field THz output from the plasma with parabolic mirrors and extending the probe beam such that the THz and optical probe could be matched collinearly. Additionally, a THz EOS system was also constructed by adding a Zinc Telluride (ZnTe) crystal at the common focal plane of the optical probe and THz. In Fig. 27, the signals corresponding to the EOS, conventional ABCD, and plasma OBCD are plotted together. Table 1 shows the values gathered for SNR, DR, detection bandwidth, and probe energy requirements. It is shown that the plasma OBCD provides a significant improvement over the conventional ABCD in terms of the required probe energy, SNR, and conversion efficiency. It should be noted that while the detection bandwidth in the plasma system is greater than the conventional ABCD in this experiment, the primary reason for this is that the ABCD system was not purged of water vapor. As such, the long propagation paths in the system led to the diminishing of the higher spectral frequency components. In the EOS system, the limited bandwidth is due to the phonon absorption at 3 THz in ZnTe crystals. Compared to the EOS system, the SNR in the plasma system was higher, but the DR and low probe energy requirements of the EOS system are superior.

**Table 1:** Comparison of EOS, conventional ABCD, and plasma OBCD systems in characterizing the same plasma source

System	EO sampling	Conventional TFISH	This work
SNR	300	200	400
DR	> 10^4^	10^3^	10^3^
Efficiency	N/A	10^−9^%	0.02%
Bandwidth	2.5 THz	5 THz	> 12 THz
Probe energy required	< 10 nJ	> 100 µJ	< 20 µJ

### Summary

In summary, this section presents the findings of the first TFISH system probing plasma directly. The large probe to TFISH conversion and very easily visible TFISH signals are impactful because they can lead to single-shot TFISH and ABCD/OBCD measurements without the need for ultra-sensitive and cooled CCD. Additionally, the system can work as a plasma diagnostic tool in large-scale laser facilities. In future studies, the localized THz field strength, localized plasma density, and plasma shape will be gathered in a single measurement. Moreover, the fact that these measurements are done in air means that there is no phonon interaction limiting the detection of the THz source. A deterministic separation between TFISH and charge induced EFISH remains a challenge, but the polarization properties of the single-color and two-color experiments are used to establish a clear relationship between the THz and the SH signal.

## Terahertz field induced second harmonic generation at 40° incidence

This section introduces the TFISH system evaluated at different noncollinear angles *θ*_C_. The recovered signal, polarization, and temporal dynamics are used to compare the signal to that achieved in Sect. 2.

### Experimental setup

**Fig. 28:**
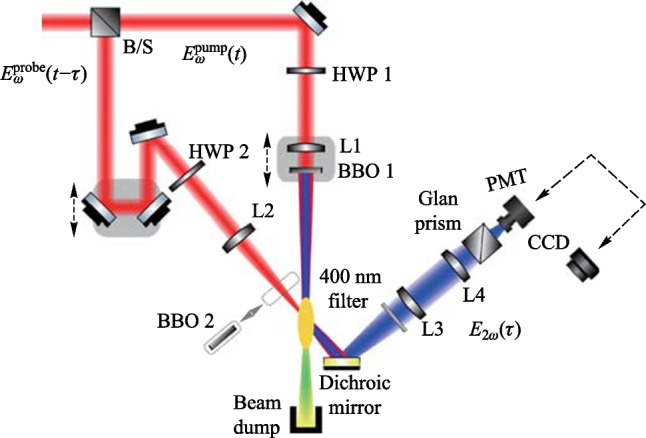
Experimental setup for the 40° TFISH experiments. The system is a two-color air plasma THz generation system where a β-BBO (BBO 1) crystal is placed after lens L1 and generates THz and plasma at the focal plane. A lens is used to focus the probe into the plasma at an angle of 40° with respect to the pump propagation direction

As shown in Fig. 28, the noncollinear TFISH system consists of a two-color air-plasma THz source where the probe beam intersects the plasma at 40° incidence. A 6.5 W and 1 kHz Coherent Astrella amplified laser system operating near 100 fs is used. The laser fundamental wavelength is 800 nm (*ω*), and the initial beam spot size is measured to be 12 mm 1/e^2^. The pump path contains 5.2 W while the probe contains 1.3 W. A 100μm thick type I β-Barium Borate (BBO 1) crystal is placed between the pump lens and the plasma to maximize the far-field THz yield. The two-color plasma is produced by a 300 mm lens (L1). The focal length used in this experiment is much larger than that in Sect. 2 to form a longer filament while allowing enough space for the probe to mix into the plasma.

A conventional 150 mm lens (L2) is used to focus the probe beam onto the pump plasma. Next, a 4F imaging Keplerian telescope consisting of a 75 mm lens (L3) and a 150 mm lens (L4) is used to image the plasma interaction to the detection plane. The detector used is either a photomultiplier tube (PMT) or a conventional CCD (Imaging Source DMK 27BUP031). A dichroic mirror and a combination of two bandpass filters (40 nm FWHM centered at 400 nm) are used to separate the SH spectrally and spatially from the probe *ω*. The pump frequencies narrowly miss the dichroic mirror and are not detected in the experiment. The polarization of the pump and probe beams are controlled with half wave plates (HWP) HWP 1 and HWP 2, respectively. Lastly, a Glan–Thompson prism is used as an analyzer for the SH.

Under normal operation, the SH produced by the system follows the same formalism as the TFISH process described in Sect. 2. Therefore, the signal is also described as a four-wave mixing (FWM) operation [37]:

13 $${S}_{2\omega }\propto {\int }_{-\infty }^{\infty }{\left|{\chi }_{ }^{\left(3\right)}\left(2\omega ,\omega ,\omega ,{\omega }_{\mathrm{THz}}\right){{E}_{\omega }}^{2}\left(t - \tau \right){E}_{\mathrm{THz}}^{*}\left(t\right)\right|}^{2}\partial t,$$

where *S*_2*ω*_ is the detected PMT signal, ${\upchi }^{\left(3\right)}\left(2\omega ,\omega ,\omega ,{\omega }_{\mathrm{THz}}\right)$ is the third-order susceptibility tensor of air within the plasma, ${E}_{\omega }\left(t \pm\uptau \right)$ is the electric field of the probe beam delayed by a temporal factor* τ*, and ${E}_{\mathrm{THz}}\left(t\right)$ is the THz field confined to the plasma filament. The main motivation for realizing the system away from 90° is to be able to design a single-shot system using spatio-temporal couplings (STC). As briefly discussed in Sect. 1, the single shot capability of a THz detection system depends on either angular dispersion or pulse-front tilt (PFT). The easiest STC to implement is PFT because it is the most widely used in state-of-the-art single-shot electro-optic sampling (EOS) systems [49]. However, for PFT, the optical beam used is typically collimated onto the interaction region. In a 90° incidence system, this is an issue because not only are the temporal overlaps for the beams mismatched along the propagation direction, the interaction of the probe with the plasma is spatially limited and would lead to a significant loss in the TFISH signal. At angles close to a collinear geometry, the interaction can be maximized, and it can be ensured that the beams are (when neglecting the PFT) co-timed throughout their radii. This allows for a pump–probe experiment to be possible with only the PFT providing a temporal mismatch.

### Results and discussion: noncollinear TFISH analysis

**Fig. 29:**
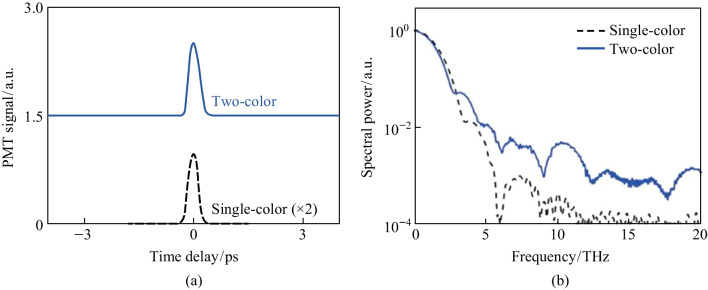
**a** Temporal TFISH signals gathered for two-color (solid line) and single-color *ω* (dashed line) pumps. The waveforms are vertically shifted for clarity. **b** Spectra corresponding to the TFISH signals. The signals are gathered in multi-shot operation

In Fig. 29a, the temporal TFISH profiles for the two-color and single-color pumped THz sources as a function of temporal delay for the optimized position along the plasma are shown. The power spectrum is revealed in Fig. 29b by performing a Fourier transform operation on the TFISH signals. The spectrum appears as a conventional TFISH spectrum and spans a broad spectral detection range as expected.

**Fig. 30:**
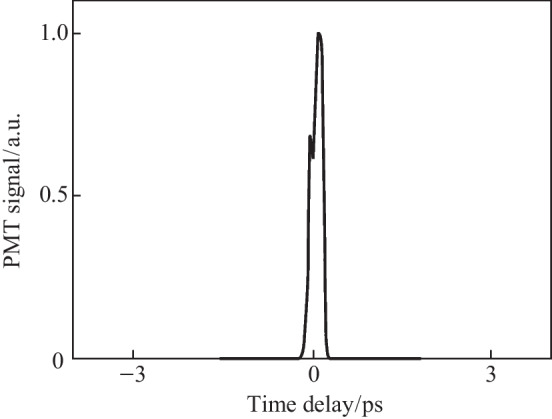
More sensitive detection of a two-color signal as shown in Fig. 29a when a shorter focal length lens is used. The phase reversal point at time delay *τ* = 0 is made visible. Further alignment and optimization would improve the signal

The maximized TFISH signals in the 40° system are within the same order of magnitude as the signals gathered from the 90° system. In the multi-shot operation, the TFISH signal recovered at the PMT for two-color pumping reaches 4 μW when a probe beam < 10 mW is used, leading to a probe *ω* to SH energy conversion efficiency > 0.04%. This conversion efficiency is nearly double the conversion efficiency found in the 90° system. The enhanced conversion is attributed to the longer interaction region between the pump and probe. The single-color system requires a larger probe power at 100 mW to reach its maximum signal. Thus, the single-color conversion efficiency is on the order of 10^−4^%. Crucially, when either the probe beam or the pump beam is blocked independently, the TFISH signal disappears. The signal shown in Fig. 29a is also noticeably different from the signal in Fig. 6a in that it is not as sensitive to the modulation depth and the point of phase reversal is not easy to see at delay time *τ* = 0. When a shorter focal length is used and more careful alignment is done, the system becomes more sensitive and a signal like that shown in Fig. 30 is seen. In Fig. 30, the 150 mm probe lens is replaced with a 50 mm lens to recover a more sensitive TFISH signal. While it is clearly beneficial to use the shorter focal length lenses for the probe, all further experiments will continue to use the 150 mm lens for two reasons: (1) using a short focal length increases the focal intensity which leads to faster ionization; and (2) to procure coherent signals via the optically biased coherent detection (OBCD) method while avoiding damage to the BBO 2 crystal, a longer probe focal length is needed.

#### Polarization characterization for single color air plasma

**Fig. 31:**
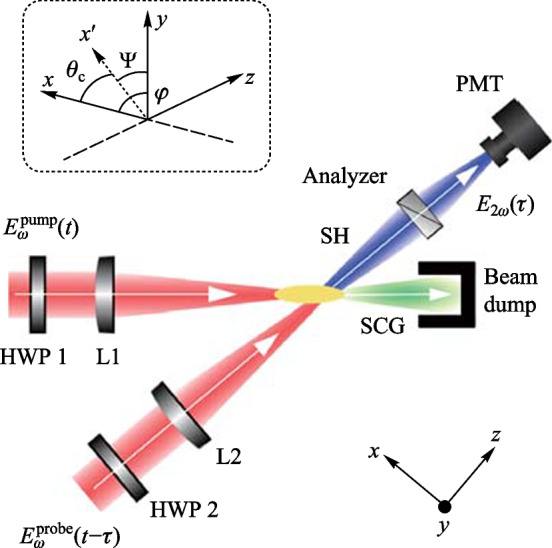
Simplified schematic of Fig. 28 for the polarization characterization system in single-color air plasma. The *z*-axis is defined along the propagation direction of the probe beam. The inset shows the detailed orientation of the pump and probe as they are defined from the *x*-axis and* x′*-axis, respectively

Next, polarization is characterized. In Fig. 31, a simplified system schematic is shown that focuses on the involved planes of polarization. By removing BBO 1 from the system, the single-color polarization dependence is characterized. Like in the 90° system, the probe polarization is characterized by an angle *φ* in the probe beam *x*–*y* plane measured from the *x*-axis. The cartesian planes are defined so that the p-polarized component of the probe lies on the *x*-axis. The pump in the 40° system is projected along the *x′*-*y* plane where a new axis $x^{\prime} = x\mathrm{cos}{\theta }_{\mathrm{C}} - z\mathrm{sin}{\theta }_{\mathrm{C}}$ is defined given *θ*_C_ = 40°. The pump polarization is measured from the *x′* axis and has a value Ψ = 0° and Ψ = 90° to represent pump p and s-polarization respectively. The probe power used is kept at 100 mW for all experiments.

**Fig. 32:**
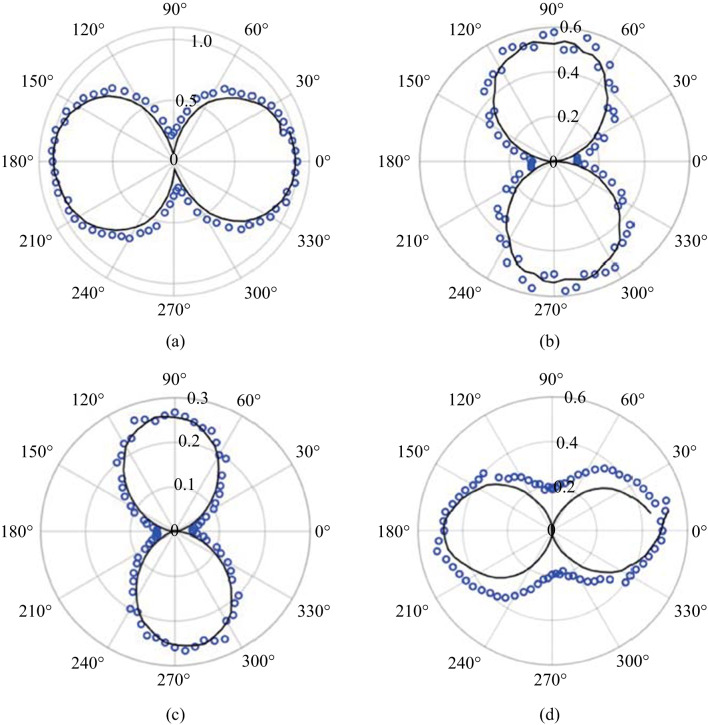
Characterization of the SH using a Glan analyzer. Blue circles represent real data gathered in experiment. The data are normalized to the highest value for the following pump-probe permutations: **a** p–p, **b** p–s, **c** s–p, and **d** s–s. For each data set, the black lines are the normalized experimental data with respect to a range [0 1]

The polarization permutation notation is taken as pump-probe polarization. In Fig. 32, the characterization for each of the four permutations is shown. Like in the 90° system, the polarization of the SH neither follows the pump or probe explicitly. Rather, the SH polarization is elliptical with its major axis along p for permutations where the pump and probe polarizations are the same (p–p and s–s) and with the major axis along s when the pump and probe are orthogonally polarized. In Fig. 32, the permutation plots are normalized with respect to the largest signal. The blue circles show the real data gathered from the experiment while the black lines represent the normalized values for each “peanut plot” gathered by normalizing to a range between 0 and 1.

The black lines aid in determining the overall orientation of the SH polarization. Interestingly, unlike in the 90° system where the s–s permutation was the optimum configuration for the SH, the optimum configuration in the 40° system is the p–p configuration. The other configurations are weaker, but the s–s configuration is surprisingly weak—only reaching 50% of the p-p signal. Given that the s-components of the polarization are always in-plane as shown in the inset of Fig. 31 and Eq. (3), the reason for the reduced signal in the s–s is not currently known.

**Fig. 33:**
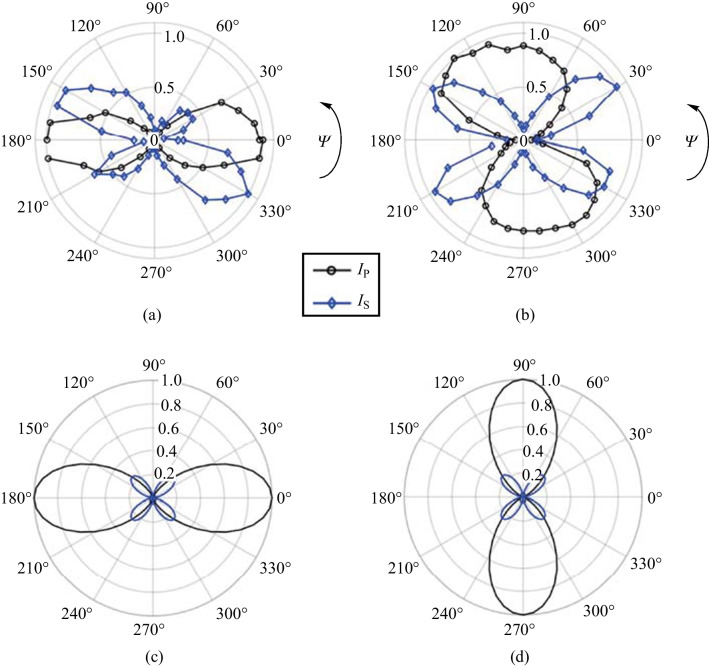
**a** Experimental data plot of the s-component and the p-component of the TFISH 2*ω* as a function of pump beam polarization from Ψ = 0° to Ψ = 360° while *φ* = 0°. **b** Experimental data plot of the s-component and the p-component of the TFISH 2*ω* as a function of pump beam polarization from Ψ = 0° to Ψ = 360° while *φ* = 90°. The black circles represent the p-component SH while the blue diamonds represent the s-component of the SH. **c** Rotating linear polarizer simulation result for an input p-polarized state onto a rotating p-pass polarizer. **d** Rotating linear polarizer simulation result for an input p-polarized state onto a rotating s-pass polarizer

In Sect. 2, the violation of Eq. (2) and the fact that the polarization of the SH did not follow either the pump or probe explicitly was used to neglect the *χ*^(2)^ contribution of the plasma in generating the SH. The apparent switching between s- and p-polarized SH was shown to be attributed to a radial electric field which manifested in experiments as an asymmetry in the SH components with respect to the probe and pump polarization rotation. Here, a similar effect is shown in Fig. 33. However, unlike in the 90° system, when the pump or probe is set to p-polarization while the other beam is rotated, the p-component of the SH is maximized along the horizontal axis. In Fig. 33a the pump polarization is rotated from 0° to 360° while the probe polarization is kept static at p-polarization. The twofold and fourfold symmetry dependence of the p- and s-components of the SH are gathered by setting the Glan prism to pass either s- or p-polarization, respectively. Again, it is clearly seen that when the pump and probe are co-polarized at p-polarization (*φ* = Ψ = 0°), the magnitude of the horizontal component is dominant leading to the larger signal when the p–p permutation is used in Fig. 32a. When *φ* is set to 90° (Fig. 33b), the polarization asymmetry is maintained and looks like the patterns seen in Fig. 15a. Figure 33c, d show simulation results of treating the plasma as a rotating linear polarizer as discussed in Sect. 2. For Fig. 33c, the input state is a p-polarized state onto a rotating linear polarizer set to pass p-polarization when at the Ψ = 0° of its rotation. Figure 33d shows the case for an input p-polarized state onto a rotating linear polarizer set to pass s-polarization when at the Ψ = 0° of its rotation. The overall dependence on the rotation of the polarization between the experimental data and the simulated model agree very well although the magnitude of each component (s and p) do not. The trend of the data, as shown in Sect. 2, is indicative of a radially polarized field involved in a *χ*^(3)^ process such that only EFISH/TFISH processes need to be considered.

Moreover, recent work published by Mou et al. in 2023 has explored a similar polarimetry procedure to characterize polarization of arbitrary THz beams using TFISH methods [138]. Their method, termed THz Unipolar Polarimetry (TUP) uses the symmetry conditions of *χ*^(3)^ processes to infer the polarization of the THz based on the SH wave polarization. When TUP analysis is applied to this work, the source evaluated in Fig. 28 is shown to exhibit a radial polarization profile.

#### Polarization characterization for two-color air plasma

**Fig. 34:**
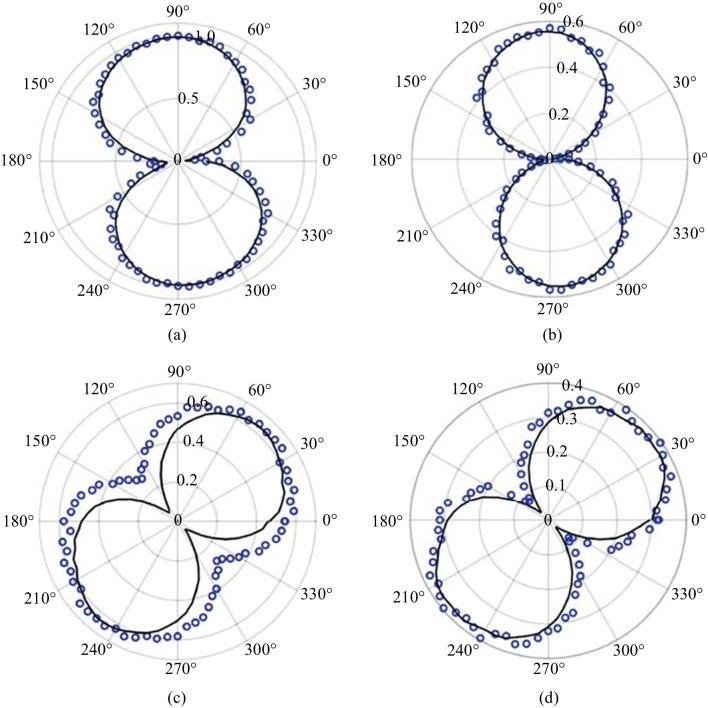
Polarization characterization of the TFISH intensity using two-color pumped air plasma in the 40° system. The blue circles represent the real data measured in the experiment by rotating the Glan prism for a given pump-probe permutation. The black lines represent the data normalized between 0 and 1 to highlight the major axis of the polarization of the SH. The permutations shown are: **a** p–p, **b** p-s, **c** s–p, and **d** s–s

The polarization characterization for the two-color pumped plasma is discussed next. The BBO 1 crystal is placed in front of lens L1 to produce a two-color THz field confined in the plasma filament. The BBO 1 crystal is rotated along with the pump polarization to control both, the pump *ω* and pump 2*ω* polarizations. When the crystal is set so that the fast axis (extraordinary axis) is horizontal, the pump polarization incident orthogonal to the fast axis leads to linear SH produced aligned with the fast axis. Because the system produces the strongest 2*ω* orthogonal to the *ω* beam in this configuration, the crystal can be rotated about its azimuth so that the pump polarization becomes elliptical with components along the pump 2*ω* polarization axis. The results of the polarization characterization are shown in Fig. 34 for permutations designated as *ω* pump-probe. Like in the 90° system, the polarization of the TFISH is preferentially characterized with a major axis along s-polarization. However, the analysis for the polarization in the 40° system is more complex because after the pump BBO 1, the *ω*-pump polarization can take elliptical polarization as opposed to the linear modes analyzed in Sect. 2. Further, the longer interaction length with the plasma can allow for rotation and deformation of the polarization [125, 133].

#### Two-color OBCD and plasma characterization

The interferometric mixing of the TFISH signal with a controlled second harmonic then provides a coherent THz signal. Using the BBO 2 crystal as seen in Fig. 28, the 2*ω* (400 nm) probe is termed a Local Field-Induced Second Harmonic (LFISH) generated wave. Considering that some of the *ω* probe beam propagates unconverted, the mixing of the two SH signals has the form:

14 $$\begin{array}{c}{S}_{2\omega } \propto {\left|{E}_{2\omega }^{\mathrm{LFISH}} + {E}_{2\omega }^{\mathrm{TFISH}}\right|}^{2} .\end{array}$$

**Fig. 35:**
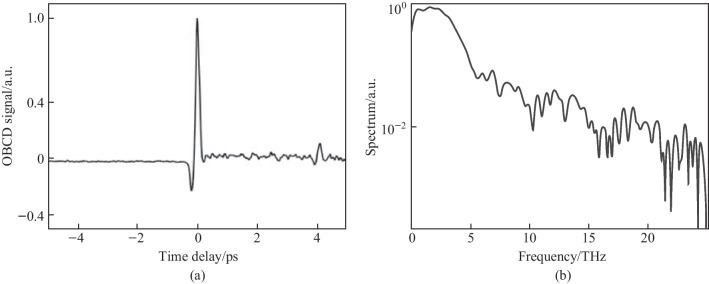
**a** Temporal OBCD signal gathered in multi-shot operation. The signal is gathered for a p-polarized *ω* pump onto BBO 1 set 135° from the fast-axis horizontal and a p-polarized *ω* probe onto BBO 2 set 45° from the fast axis horizontal. **b** Spectral power obtained from the Fourier Transform of the temporal trace plotted in log scale

The evaluation of the above expression produces two incoherent intensity traces and a cross-correlated OBCD term. The coherent signal is shown in Fig. 35a mostly isolated from the background LFISH term through lock-in detection, and its corresponding spectrum is shown in Fig. 35b after Fourier transform operation. It should be noted that because the plasma also partially modulates the probe beam, the background may not be fully eliminated and can lead to the emergence of DC components in the spectrum.

Interestingly, Fig. 35a shows a pulse at delay *τ* = 4 ps succeeding the initial signal at *τ* = 0. Because as shown in Fig. 28, there are no optical elements or relaying components between the generation and detection of THz, a reflection of the THz wave cannot explain the secondary pulse. Recent progress in TFISH systems by Bodrov et al. in 2020 has shown that when a TFISH experiment is conducted in a *χ*^(3)^ material, the SH is not produced in the bulk of the material, but rather, in the interfaces of changing *χ*^(3)^ [139, 140]. Using a 3 mm thick fused quartz plate, Refs. [139, 140] show that time delay data can be used to identify materials and hidden layers within materials. Here, the mechanism of TFISH is used to estimate the electron number density.

The focus of the probe beam is placed such that the geometrical focus is on the center of the pump plasma. In Fig. 35a, the delayed pulse near 4 ps is measured by the delayed probe as the THz wave catches up while exiting the filament. Using this figure, the length of interaction in the plasma is given geometrically by the expression *L*_d_. From Fig. 35b, the peak THz frequency is found to be 1.6 THz which corresponds to a wavelength of 187.5 μm. It is expected that the plasma will absorb and screen the THz [25]. The absorption will manifest as the peak ratio of the two signals in Fig. 35a. The electron density can be estimated by using the expressions [141]:

15 $${\alpha }_{\mathrm{loss}} = \frac{2{\omega }_{\mathrm{THz}}\kappa }{c} = -\frac{2}{{L}_{\mathrm{d}}}\mathrm{ln}\left(\frac{{A}_{2}}{{A}_{1}}\right),$$

16 $$\kappa = \sqrt{\frac{\sqrt{{\mathfrak{R}\left\{\varepsilon \right\}}^{2}+{\mathfrak{I}\left\{\varepsilon \right\}}^{2}}- {\mathfrak{R}\left\{\varepsilon \right\}}^{2}}{2}},$$

17 $$\varepsilon = 1 - {\omega }_{\mathrm{p}}^{2}\left[\frac{{{\tau }_{\mathrm{s}}}^{2}}{1+{{\omega }_{\mathrm{THz}}}^{2}{{\tau }_{\mathrm{s}}}^{2}} +\mathrm{j}\frac{{{\tau }_{\mathrm{s}}}^{2}}{{\omega }_{\mathrm{THz}}{\tau }_{\mathrm{s}}\left(1+{{\omega }_{\mathrm{THz}}}^{2}{{\tau }_{\mathrm{s}}}^{2}\right)}\right],$$

where *α*_loss_ is the absorption coefficient in m^−2^, *κ* is the extinction coefficient, *A*_1_ is the OBCD amplitude at time *τ* = 0, *A*_2_ is the signal amplitude at *τ* = 4 ps, *ε* is the complex dielectric function, *ω*_p_ is the electron plasma frequency, and *τ*_s_ is the electron scatter time. Assuming a scatter time of 1 ps in a clamped fs filament [25] and using the $\sim 9$ times ratio between the two waveform peaks, the estimated electron number density is on the order of 10^16^ cm^−3^ as previously found in Sect. 2. This method unfortunately cannot return localized electron density because of the large interaction region. The TFISH method proves to be useful in characterizing the plasma because although the critical density at THz frequency is close to the plasma density, the produced SH has a critical density many orders of magnitude larger than the electron density and is thus allowed to propagate through the filament with minimal absorption or scattering.

### Potential for single-shot systems

The configuration in this section is further increased in complexity due to the pulsed nature of the beams. The system can then be referred to as a simulated pulse-front tilt (SPFT) system because when considered with respect to the pump propagation axis, the probe arrival time varies along the transverse plane. In other words, the plane of the arrival of the intensity in the probe appears tilted with respect to that of the pump. Because the system includes an SPFT, there is temporal information encoded onto the extent of the probe beam—parts of the probe beam cross and exit the interaction region while others have not arrived at the interaction region at a given time *t*. While this makes the system less sensitive, it means that a single-shot system could be devised using only the angle *θ*_C_. The PMT is removed and replaced with a conventional CCD to capture the full trace of the probe beam.

**Fig. 36:**
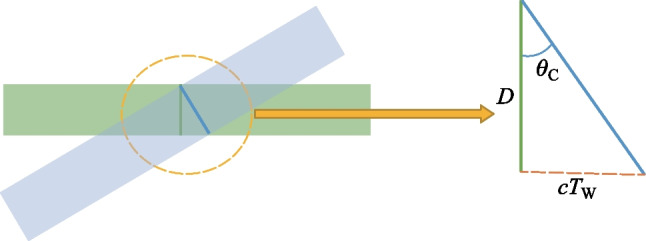
Geometrical Representation of the simulated pulse-front tilt (SPFT) showing the probe (blue) and pump (green) beams along the focal interaction region. The beams are considered within the Rayleigh range so that the beam phase fronts are essentially plane. Above, the bottom of the probe beam leads the top of the probe beam. Where the apex of the triangle indicates interaction where the pump and probe arrive at the same time, the base of the triangle highlights an event where the pump is delayed from the probe. The positioning between the two planes of constant intensity can be controlled with an additional mechanical delay stage. The interaction diameter—limited by the THz and the probe beam diameter—is signified with variable D and the distance between the phase fronts along the radial coordinate is given as *cT*_W_. A geometrical analysis can reveal the expression for the SPFT time window

The inspiration for single-shot analysis in the SPFT system comes from work done in Ref. [47] in electro-optic sampling (EOS) systems by Shan et al. in 2000. The basis for single-shot detection relies on the spatial overlap between the ω probe and the THz beam being imaged to the CCD. Because the probe impinges on the beam at an angle of 40°, the overlapped spatial region directly yields a temporal coordinate through Fig. 36. Where the apex of the triangle indicates interaction where the pump and probe arrive at the same time, the base of the triangle highlights an event where the pump is delayed from the probe. The positioning between the two planes of constant intensity can be controlled with an additional mechanical delay stage. A geometrical analysis of the phase fronts and intensity fronts yields the expressions ${T}_{\mathrm{W}} = \frac{D\mathrm{tan}\theta }{c}$ and $\Delta t_{R} = \frac{\Delta x\tan\theta }{c}$, representing the temporal window and device-limited resolution, respectively. In the above expression, *T*_W_ represents the spatio-temporally coupled total detection time window, *D* is the optical beam diameter, *θ* is the angle between the pump and probe, Δ*t*_R_ is the temporal resolution per pixel on the CCD, and Δ*x* is the CCD pixel width.

It is important to note two crucial limits in the above system: (1) The resolution can never truly be less than the optical short pulse duration since it is the measuring tool used in the actual experiment, and (2) aside from the angle, the diameter of the probe and pump will dictate the true detection time window. Point (2) is crucially important because for conventional focusing, the probe beam is much smaller than the plasma at approximately < 20 µm diameter. Unfortunately, the dependence of the noncollinear TFISH signal on the intensity and the dependence of the timing window on the probe diameter are diametrically opposed and lead to a trade-off. When the interaction region is directly imaged to the CCD, integrating over the row of pixels in a CCD image leads to the detection of the TFISH signal due to the linear relationship between *T*_W_ and *D*. This limits the THz detection within a time window of 55 fs, which is much smaller than the THz pulse duration estimated from Fig. 35a. To improve the detection, either the angle should be increased, or the probe beam diameter should be increased.

**Fig. 37:**
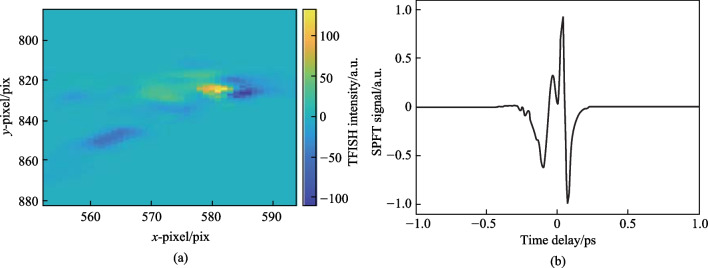
**a** SPFT OBCD CCD image after background subtraction. **b** Retrieved TFISH trace from the CCD image

A typical single-shot image is shown in Fig. 37a for the OBCD system. To retrieve the temporal trace, the interaction region is imaged to the CCD plane. A computer analysis of the image is done while averaging over every row of pixels. In Fig. 37b the retrieved SPFT signal is shown in the 40° system by slightly moving the probe away from focus to retrieve a larger probe beam diameter. This essentially reduces the detection capability of the TFISH (defocusing reduces the probe intensity which therefore reduces the TFISH signal) but allows for a larger detection time window. The resulting trace is calibrated and shows a resolution of 8 fs/pix and a total usable time window of 220 fs. The resolution is experimentally measured by gathering the single-shot signals at two different delay stage positions and is close to the theoretical 6 fs/pix resolution expected from the SPFT expression. The resolution in fs/pix is the ratio of the time difference between the two stage positions over the number of pixels that the peak of the signal travels between the two stage positions. The CCD exposure time is set to 1 ms corresponding to an average operation over a single pulse (a single shot) and the trace is further attenuated to avoid saturation at the CCD.

In the SPFT system, the signal appears distorted because the trace is limited by the small probe beam diameter which leads to loss of spectral information. The limited size of the probe beam near 80 μm leads only to a maximum usable window of 220 fs. While the window was vastly improved from the focused beam case (4 times improvement), it is still shorter than the previously measured THz pulse duration $\sim$ 300 fs. This creates a strong windowing effect for the much longer THz pulse, making it very difficult to retrieve the true shape of the signal. Defocusing the beam more or using a cylindrical lens can help in increasing the probe beam diameter and therefore increasing the detection time window, but the exposure of the CCD would need to be increased such that the system is no longer operating in a single shot.

### Summary

In this section, it was shown that the system at 40° could also produce a strong SH that can be described by an EFISH/TFISH process. The polarization was characterized in the single-color and two-color modes and the OBCD was again used to develop a method of characterizing the plasma and specifically estimate the averaged electron number density in the plasma. Additionally, EOS techniques were applied to develop and test the capability of producing a single-shot TFISH/OBCD system. While the single-shot signal suffered from windowing effects and was very distorted, the results were promising in that they show that single shot EOS techniques can be readily applied in TFISH/nonlinear detection systems to produce broadband single shot THz detectors.

## Single-shot detection of terahertz field induced second harmonic generation

This section presents the theoretical and experimental development of a single shot terahertz (THz) detection system. The principles of single shot electro-optic sampling (EOS) are applied to terahertz field-induced second harmonic (TFISH) generation to conceptualize a single shot broadband detection system. This section builds upon the concepts explored in the previous sections—especially the polarization and use of the optically-biased coherent detection (OBCD) methods—to produce a single shot trace that is visible to a conventional CCD (Imaging Source DMK 27BUP031).

It should be noted that an experiment like what is presented in this section was shown by Nomura et al. in 2013 [142]. A two-color air plasma source was used to produce mid-wave infrared (MWIR) and far infrared (FIR) radiation. A combination of frequency-resolved optical gating (FROG) and electric field-induced second harmonic (EFISH) generation was then used to characterize the MWIR and FIR radiation. The system was also shown to operate in a real-time scheme (the optical probe was too weak to produce a true single-shot signal) with a maximum temporal window near 300 fs. However, the radiation presented in Ref. [142] and in the group’s following papers did not show any THz components as defined in Sect. 1 (between 0.1 and 10 THz). In fact, the radiation presented was constrained to a region between 10 and 30 THz (and with a peak frequency near 15 THz). The FROG signal also only included higher frequency components with a center near 90 THz. A potential reasoning for the lack of frequencies in the THz regime could be that due to the low pump optical power, the generated THz is too weak to be detected—especially by TFISH and real-time detection methods. Second, although not mentioned, a set of filters could be used to block low frequency components since the goal in Ref. [142] is to produce phase-stable MWIR pulses rather than THz. Regardless, because no THz is detected, the work presented in this section is (to the best of our knowledge) the first example of THz single-shot and real-time detection using TFISH methods from plasma. Additionally, the signal shown in this section boasts a larger detection time window and single-shot capability.

The typical conversion efficiencies achieved in conventional multi-shot TFISH experiments are on the order of 10^−9^% [37, 39]. This makes constructing a single shot TFISH or OBCD system very difficult because a 1 W probe beam would only produce a 10 pW second harmonic (SH) signal. A very sensitive and cooled CCD would be needed to view the beam even in a focused geometry. However, most single shot methods are based on pulse-front tilt (PFT) and require the probe to be collimated (at least along one dimension) to produce a single shot signal [49]. Because the TFISH process is dependent crucially on the square of the probe intensity, the SH signal would be imperceptible with a conventional CCD. The work done in Sects. 2 and 3 showed that the conversion efficiency of the TFISH ω probe to SH could be vastly improved by either using a material with different *χ*^(3)^ or by spatially confining the THz in the filament to achieve a higher peak electric field strength. With conversion efficiencies more than five orders of magnitude larger than the conventional systems, the single shot PFT methods used in single shot EOS systems are realizable in the TFISH system.

### Experimental setup

**Fig. 38:**
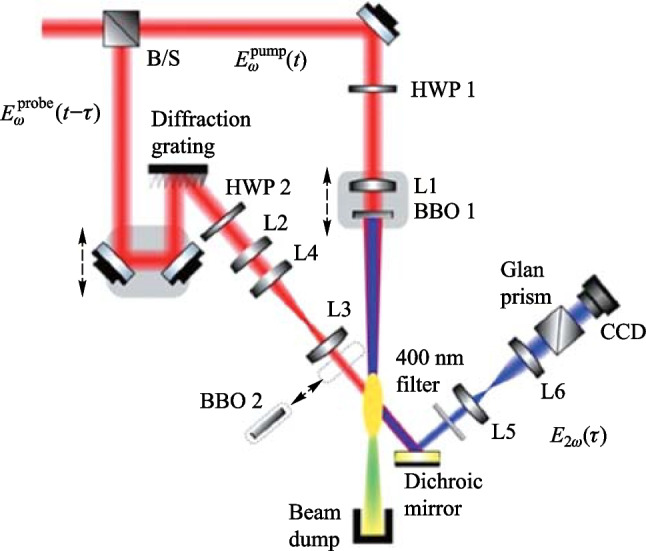
Schematic of the single shot TFISH system using PFT. The system is a two-color air plasma THz generation system where a β-BBO (BBO 1) is placed after lens L1 and generates THz at the focal plane. A grating is used to induce a PFT on the probe. The pulse-front tilt is then imaged onto the interaction region by a Keplerian telescope. A CCD is used to detect the SH generated. The green color represents the supercontinuum generated from the pump plasma

As shown in Fig. 38, the noncollinear TFISH system consists of a two-color air-plasma THz source where the probe beam intersects the plasma at 40° incidence like the system shown in Sect. 3. A Coherent Astrella amplified laser system operating at 6.5 W, 1 kHz repetition rate, and near 100 fs is used. The laser fundamental wavelength is 800 nm (*ω*), and the initial beam spot size is measured to be 12 mm at 1/e^2^. The pump path contains 5.2 W while the probe contains 1.3 W. A 100 μm thick type I β-Barium Borate (BBO 1) crystal placed between the 300 mm pump lens (L1) and the plasma to maximize the far-field THz yield.

An 800 lp/mm grating is used to produce a PFT of 40° along the intensity-front of probe with respect to its wavefront. The PFT is manifested as a time to space mapping where it can be used to encode temporal information onto the spatial coordinate. This configuration is hereby referred to as a single shot/real-time PFT system and has its PFT directly imaged from the grating to the focal region with a Keplerian telescope consisting of a set of cylindrical lenses with focal lengths of 300 (L2) and 100 mm (L3). This is considered a “Grating-Imaging system”. Importantly, the PFT is designed so that its projection onto the pump when considering the noncollinear interaction angle leads to a larger PFT than 40°. Because the PFT probe is effectively collimated in the interaction region between the probe and the plasma, a third cylindrical 200 mm lens (L4) is placed between the cylindrical lenses in the telescope to focus the beam along the direction orthogonal to the induced PFT (vertical axis). The result is a higher intensity along a line in the focal region. In both configurations, the interaction is then finally imaged to a CCD by a Keplerian telescope using spherical lenses. A combination of filters and dichroic mirrors is used to discriminate the TFISH signal from the fundamental probe.

To achieve coherent detection, a second 100 μm thick type I β-Barium Borate (BBO 2) is used along the probe beam path to produce a reference wave. When the polarization of the SH wave induced by BBO 2 matches or has components along the same axis as the polarization of the TFISH signal, an interferometric process leads to coherent homodyne detection of THz via OBCD. The polarization is controlled through rotating a half-wave plate (HWP) along the probe prior to BBO 2 and by rotating the crystal itself. A delay line is added to the probe beam path to aid in system calibration.

### Spatio-temporal coupling and Kostenbauder matrix treatment

The theoretical treatment of the PFT system requires modeling the linear propagation of an electric field $E\left(x,t\right)$ through the grating imaging system. Note that because the PFT is only induced along one transverse dimension, only either the *x* or *y* coordinate is needed. The simplest method to properly account for the numerous spatio-temporal distortions induced by the grating is to employ the Kostenbauder matrices (K-matrices) [143].

#### K-matrix formulation

The TFISH system K-matrix at the interaction region is found according to Fig. 38 as

18 $${K}_{\text{int}} =\left[\begin{array}{cccc}\frac{{f}_{3}}{{f}_{2}\sigma }& 0& 0& 0\\ 0& \frac{{f}_{2}\sigma }{{f}_{3}}& 0& - \frac{{f}_{2}\lambda \xi }{{f}_{3}c}\\ - \frac{\xi }{c\sigma }& 0& 1& 0\\ 0& 0& 0& 1\end{array}\right],$$

where *f*_2_ and *f*_3_ are the focal lengths of lenses L_2_ and L_3_, respectively. Also, $\sigma = \frac{\mathrm{cos}{\theta }_{i}}{\mathrm{cos}{\theta }_{d}}$ is the angular grating magnification, $\xi = \frac{\mathrm{sin}{\theta }_{i}+\mathrm{sin}{\theta }_{d}}{\mathrm{cos}{\theta }_{d}}$ is the grating angular dispersion parameter, and *c* is the speed of light in vacuum. The simplification used above mirrors that used in Ref. [144]. Note that L_4_ is omitted since it has no effect in the direction of the PFT or the manifestation of any STC.

To properly model the Gaussian spatio-temporal field, a reduced *Q*-matrix treatment is necessary. This is accomplished by taking the originally spatio-temporally undistorted Gaussian prior to the grating to have a form [52, 145, 146]:

19 $${E}_{x}\left(x,y,z\right)E\left(t\right) = {\mathrm{e}}^{- \frac{\left({x}^{2} + {y}^{2}\right)}{{W}^{2}\left(z\right)}}{\mathrm{e}}^{-\mathrm{j }{n}_{\mathrm{o}}{k}_{\mathrm{o}}\frac{\left({x}^{2} + {y}^{2}\right)}{2\left|R\left(z\right)\right|}}{\mathrm{e}}^{- \frac{1}{2}\left(\frac{{t}^{2}}{{T}_{\mathrm{o}}^{2}} -\mathrm{ j}\frac{C{t}^{2}}{{T}_{\mathrm{o}}^{2}}\right)},$$

whe﻿re *x* and *y* are the transverse coordinates, *z* is the longitudinal coordinate, *W*(*z*) is the Gaussian beam waist radius, *R*(*z*) is the Gaussian wavefront radius of curvature, and *C* is the chirp parameter which is related to the spectral chirp by the expression $C = \frac{\eta {T}_{\mathrm{o}}^{2}}{{T}_{\mathrm{o}}^{4} + {\eta }^{2}}$. The spectral chirp is $\eta = {\phi }_{2} = \pm {T}_{\mathrm{o}}^{2}\sqrt{{\left(\frac{{T}_{\mathrm{c}}}{{T}_{\mathrm{o}}}\right)}^{2} - 1}$, with *T*_c_ representing the chirped pulse 1/e duration. Defining new parameters ${T}_{\mathrm{Q}}^{2} = 2{T}_{\mathrm{o}}^{2}$ and $\Gamma = \frac{C}{{T}_{\mathrm{Q}}^{2}}$, and understanding the four quadrants of the K-matrix system, a general expression

20 $$E\left(x,z,t\right) = {\mathrm{e}}^{-\mathrm{j}\frac{\uppi }{\lambda }{\left[\begin{array}{c}x\\ -t\end{array}\right]}^{\mathrm{T}}\left[\begin{array}{cc}\frac{1}{{Q}_{11}}& \frac{1}{{Q}_{12}}\\ \frac{1}{{Q}_{21}}& \frac{1}{{Q}_{22}}\end{array}\right]\left[\begin{array}{c}x\\ t\end{array}\right]} = {\mathrm{e}}^{\left[{x}^{2}{Q}_{xx} + 2xt{Q}_{xt} - {t}^{2}{Q}_{tt}\right]},$$

can be established to treat Gaussian beams. By inspection, when no STC is present (${Q}_{xt} = 0$), the cross-terms vanish and the real part of ${Q}_{xx}$ is purely related to the beam waist radius while the imaginary part is related to the wavefront radius of curvature. Similarly,${Q}_{tt} = -\mathrm{j\Gamma } + \frac{1}{{T}_{\mathrm{Q}}^{2}}$. This means that the real part of ${Q}_{tt}$ is related to the pulse duration while the imaginary part contains the spectral chirp information. If the system has STC, the analysis of widths requires more thought. This is because the widths gain implicit dependence due to the nature of coupled-domain optics—If a beam has angular dispersion, the spatial width will depend on the frequency spectrum. As was the case with the ABCD matrix, a new *Q*-matrix can be found by propagating the K-matrix. Starting initially with a matrix $${Q}_{\mathrm{i}} =\mathrm{ j}\frac{\lambda }{\uppi }{\left[\begin{array}{cc}{Q}_{xx}& {Q}_{xt}\\ -{Q}_{xt}& {Q}_{tt}\end{array}\right]}^{-1}$$, the *Q*-matrix at the output of a K-matrix system is

$$Q = -\mathrm{j}\frac{\uppi }{\lambda }*\mathrm{Inverse}\left({Q}_{\mathrm{out}}\right) = \left[\begin{array}{cc}{Q}_{oxx}& {Q}_{oxt}\\ -{Q}_{oxt}& {Q}_{ott}\end{array}\right],$$

where

21 $${Q}_{\mathrm{out}} = \frac{\left[\begin{array}{cc}A& 0\\ G& 1\end{array}\right] \cdot {Q}_{\mathrm{i}} + \left[\begin{array}{cc}B& \frac{E}{\lambda }\\ H& \frac{I}{\lambda }\end{array}\right]}{\left[\begin{array}{cc}C& 0\\ 0& 0\end{array}\right] \cdot {Q}_{\mathrm{i}} + \left[\begin{array}{cc}D& \frac{F}{\lambda }\\ 0& 1\end{array}\right]}.$$

For the TFISH interaction region, *Q*_*oxx*_ describes the spatial Gaussian parameters such as waist radius and wavefront radius of curvature, *Q*_*oxt*_ is the spatio-temporal cross-coupling term, and *Q*_*ott*_ describes the temporal width of the pulse. The input *Q*-matrix, *Q*_in_ (prior to the grating system), describes the initial probe beam spatial and temporal characteristics with no coupling so that *Q*_*oxt*_ = 0. Finally, the PFT is extracted from the output *Q*-matrix by the expression ${\alpha }_{\mathrm{PFT}} = \frac{\mathfrak{R}\left\{{Q}_{oxt}\right\}}{\mathfrak{R}\left\{{Q}_{ott}\right\}}$ given in units of s/m. It is often more convenient to describe PFT by the angle between the beam’s intensity front and wavefront. For that, the expression ${\theta }_{\mathrm{PFT}} = {\mathrm{tan}}^{-1}\left({\alpha }_{\mathrm{PFT}} \times c\right)$ is used instead [147, 148]. The extension of the K-matrix and the *Q*-matrix to the CCD plane from the grating plane is not explicitly shown above because the expressions for the matrix elements becomes very complicated. However, the simulations are carried out in MATLAB and Mathematica software.

**Fig. 39:**
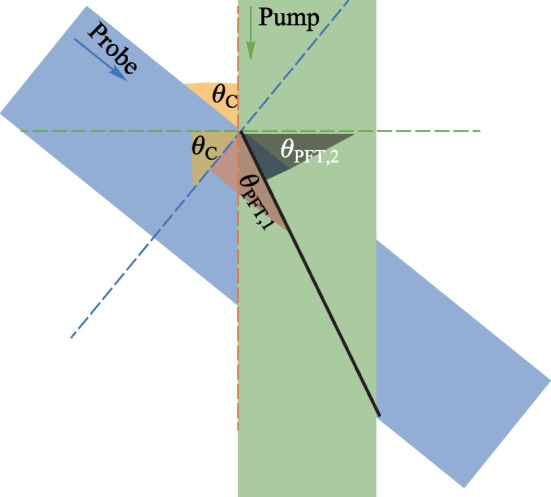
Geometrical schematic of the PFT configuration when accounting for the noncollinear angle. Taking the pump and probe beams to be within their Rayleigh lengths, the Gaussian beams can be approximated as planar. The probe beam propagates at an angle to the probe with its wavefront (dashed blue line) perpendicular to its propagation direction. The grating induces a PFT that sets the arrival of the intensity (black solid line) at an angle from the wavefront of the probe *θ*_PFT,1_. Note that the PFT is exaggerated for clarity. The arrival of the probe intensity as seen by the pump wavefront is then given by ${\theta }_{\mathrm{PFT},2}={180^{\circ}}- {\theta }_{\mathrm{PFT},1}- {\theta }_{\mathrm{C}}$

The PFT angle at the region of interaction considering the noncollinear angle is found by evaluating the system geometry as ${\theta }_{\mathrm{PFT},2}={180^{\circ}}- {\theta }_{\mathrm{PFT},1}- {\theta }_{\mathrm{C}}$, where *θ*_PFT,2_ is the PFT angle in the region of interaction, *θ*_PFT,1_ is the PFT angle imaged through the first telescope without considering the noncollinear angle, and *θ*_C_ is the angle between the pump and probe. In the interaction region, the geometry is devised according to Fig. 39. This figure shows the effect of a tilted pulse-front in addition to the angle. In the experiment, the probe beam is an ellipse with horizontal waist width radius of 1 mm and vertical waist width radius of 9 µm. The alignment is completed by first ensuring proper overlap at the focal region and then by minimizing the size of the TFISH trace. With an initial PFT of 70° and a noncollinear angle of 40°, the output PFT is 70° which means that the K-matrix simulations can be simply done without considering oblique incidences onto the interaction region (the noncollinear angle is never changed). However, when the beam is seen away from the interaction region, the spatial chirp induced by the grating is dominant and the spatio-temporal overlap leads to distorted signals.

**Fig. 40:**
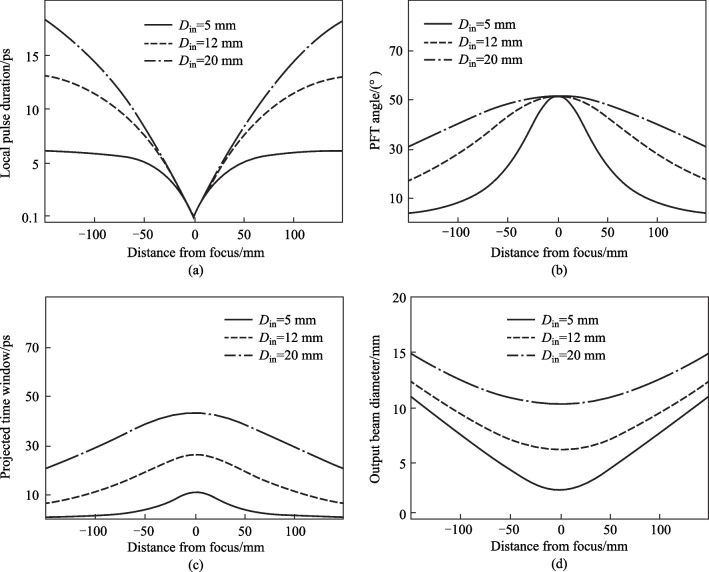
Simulation results of a K-Matrix solver for system optimization using three initial probe beam diameters. **a** Depiction of beam pulse duration lengthening because of angular dispersion as a function of distance from the CCD (lens focal plane). **b** Maximum achievable PFT vs distance from the CCD. **c** Maximum achievable time window vs distance from CCD. **d** Output beam diameter vs distance from the CCD

Because of the STC in the system, the input beam diameter is very important in determining the amount of PFT induced. Figure 40 shows the simulation of the PFT system through a K-matrix solver from the grating to the CCD while using initial probe beam diameters *D* = 5, 12, and 20 mm to find an optimized configuration. The simulation is done with a Keplerian telescope that magnifies the PFT from the interaction region to the CCD by a factor of 2 like the experiment as shown in Fig. 38. This allows for easier setup in real experiments but reduces the PFT angle from the expected 70° to 50°. As seen in Fig. 40a, the grating image plane corresponds to the only point where the pulse-duration of the probe is preserved, and spatio-temporal overlap is undistorted—leading to the maximum possible PFT angle for a given system as shown in Fig. 40b. The term “local pulse duration” refers to the fact that when the beam suffers STC, the temporal duration is dependent on the radial coordinate (*x* or *y*). In the treatment of this work, all simulated measurements are done on axis (*x* = *y* = 0) unless otherwise stated. Moving away from the focus will lead to a beam that has a larger pulse duration along the edges of the beam and a loss in the information from the correlation in Eq. (1).

In Fig. 40c, the maximum theoretically achievable time window is simulated according to the geometry in Fig. 36 and the expressions [47, 49]:

22 $${T}_{\mathrm{W}} = \frac{D\mathrm{tan}{\theta }_{\mathrm{PFT},2}}{c}\, \mathrm{and}\, \Delta {t}_{\mathrm{R}} = \frac{\Delta x\mathrm{tan}{\theta }_{\mathrm{PFT},2}}{c},$$

representing the temporal window and device-limited resolution respectively. In the above expressions, *T*_W_ represents the spatio-temporally coupled total detection time window, *D* is the optical beam diameter, Δ*t*_R_ is the temporal resolution per pixel on the CCD, and Δ*x* is the CCD pixel width. As the initial diameter onto the grating is increased, the tolerance in the positioning for achieving the maximum PFT angle and time window are relaxed; however, there remains a trade-off against the pulse duration as seen in Fig. 40a. This can be understood by noting that at the point of overlap, the spatial chirp and group delay dispersion are mostly eliminated. In this region, for an 800 lp/mm grating, this results in a theoretical tolerance of 0.6 mm.

A final consideration in setting up the experiment is the extent of the beam onto the CCD. Although the beam is collimated, the angular dispersion will cause the beam to appear divergent and elongated along the direction of the induced PFT. In Fig. 40d the simulation shows that as the beam diameter onto the grating is increased, the divergence is relaxed but yields a larger value. In our experiment, we chose a 12 mm diameter because it yielded the most relaxed parameters without having to use large aperture mirrors to guide the beam. The effect of chirping according to Eq. (21) is not significant for small values of spectral chirp. Because of this, so long as the chirp is not significantly changed during the experiment, there is no need to consider temporal chirp when selecting the PFT.

#### Resolution limitation

The true resolution in the single-shot TFISH depends not only on the CCD pixel size, but on the chirp of the probe pulse as well. The resolution is then limited by the uncertainty principle because in chirping, the method allocates a temporal marker to a specific frequency component. In considering that a pulse has an expression:

23 $${E}_{\mathrm{p}}\left(\delta \omega \right) = {\mathrm{e}}^{- \frac{\delta {\omega }^{2}}{2}\left[\frac{1}{\Delta {\omega }^{2}} +\mathrm{ j}\eta \right]},$$

where *δω* refers to the frequency detuning *ω* – *ω*_o_ and *η* is the second-order phase, the temporal system resolution can be derived. The PFT probe spectroscopy can be modeled as a Gaussian function that has a resolution given by Eq. (22) with a spectral width $\mathrm{\Delta \Omega }= 1/\Delta {\tau }_{\mathrm{R}}$. The Gaussian spectrum then defines the system transfer function:

24 $${E}_{\mathrm{s}}\left(\delta \omega \right) = {\mathrm{e}}^{- \frac{\delta {\omega }^{2}}{2\Delta {\Omega }^{2}}}.$$

If the pulse acts as an input, the output from the spectrometer is the multiplication of the input and the transfer function:

25 $${E}_{\mathrm{out}}\left(\delta \omega \right) = {\mathrm{e}}^{- \frac{\delta {\omega }^{2}}{2}\left[\frac{1}{\Delta {\omega }^{2}} + \frac{1}{\Delta {\Omega }^{2}} +\mathrm{ j}\eta \right]}.$$

To get the system resolution, a Fourier transform is performed on this output and the effective temporal width is found. This will yield the output temporal limit as given by the uncertainty principle. The temporal width can be characterized as a parameter:

26 $$2{T}_{\mathrm{p}}^{2} = \frac{2\left|{T}_{\mathrm{p}}^{2}\right|}{\mathfrak{R}\left\{{T}_{\mathrm{p}}^{2,\text{*}}\right\}} = \frac{{\left(\frac{1}{\Delta {\Omega }^{2}} + \frac{1}{\Delta {\omega }^{2}}\right)}^{2} + {\eta }^{2}}{\left(\frac{1}{\Delta {\Omega }^{2}} + \frac{1}{\Delta {\omega }^{2}}\right)},$$

su﻿ch that ${E}_{\mathrm{out}}\left(\delta \omega \right) = {\mathrm{e}}^{- \frac{{t}^{2}}{2{T}_{p}^{2}}}$. The minimum of Eq. (26) yields the resolution and is found by taking the derivative $\frac{\partial {T}_{\mathrm{p}}^{2}}{\partial \mathrm{\Delta \Omega }}$ and setting equal to zero:

27 $$\frac{\partial {T}_{\mathrm{p}}^{2}}{\partial \mathrm{\Delta \Omega }} = \frac{2\left[\frac{{\eta }^{2}}{{\left(\frac{1}{{\Delta \omega }^{2}} + \frac{1}{{\Delta\Omega }^{2}}\right)}^{2}} - 1\right] }{{\Delta\Omega }^{3}}= 0.$$

The resulting minimum resolution is approximated as

28 $${\Delta\Omega }_{\mathrm{min}}= \sqrt{\frac{{\Delta \omega }^{2}}{{\eta \Delta \omega }^{2} - 1}}.$$

In the case of zero chirp, Δ*T*_R_ will be equivalent to the optical pulse duration since *η* = 0. However, if the pulse is chirped, the minimum resolution is given as

29 $$\Delta {T}_{\mathrm{R}} = \sqrt{{T}_{\mathrm{o}}\sqrt{{T}_{\mathrm{c}}^{2} - {T}_{\mathrm{o}}^{2}}},$$

where $\eta = \pm {T}_{\mathrm{o}}^{2}\sqrt{{\left(\frac{{T}_{\mathrm{c}}}{{T}_{\mathrm{o}}}\right)}^{2} - 1} = \pm {T}_{\mathrm{o}}\sqrt{{T}_{\mathrm{c}}^{2} - {T}_{\mathrm{o}}^{2}}$. In the long chirp limit, the resolution is equivalent to the single-shot EOS angular dispersion/frequency encoding method [45] since ${T}_{\mathrm{c}} \gg {T}_{\mathrm{o}},$ so $\eta \approx \pm {T}_{\mathrm{o}}\sqrt{{T}_{\mathrm{c}}^{2}}$. The result is $\Delta {T}_{\mathrm{R}} = \sqrt{{T}_{\mathrm{o}}{T}_{\mathrm{c}}}$. In the TFISH experiments conducted in this section, the pulse duration is maintained near 100 fs. Considering that the laser has a pulse transform-limited duration near 35 fs, the system resolution is 57 fs.

Compared to the frequency encoding method, the chirp does not need to be controlled in both arms to give an acceptable resolution and time window simultaneously. This is a major reason for choosing to use PFT instead of angular dispersion in constructing the single-shot TFISH system. For example, with the PFT method, since the time window is independent of the resolution, a time window of 1 ps is realizable with a 57 fs resolution. In contrast, using the frequency encoding method leads to a minimum 187 fs resolution. It should be noted that the limitation of the chirped probe can also be solved by using a second short-pulsed probe to interact with the chirped probe. Instead of analyzing the frequency modulation explicitly, the interaction of the two probes leads to a spectral interferogram with a time window equal to the chirped pulse duration and a resolution equal to the transform-limited probe pulse duration. However, this method is much more complex than either the PFT or the angular dispersion method in Ref. [45]. This method has been used before to produce single-shot EOS systems based on angular dispersion [46].

### Results and discussion: single-shot incoherent detection of air plasma sources

The noncollinear system can be modeled using the TFISH process to retrieve a signal:

30 $${S}_{2\omega } \propto {\int }_{-\infty }^{\infty }{\left|{\chi }^{\left(3\right)}\left(2\omega ,\omega ,\omega ,{\omega }_{\mathrm{THz}}\right){{E}_{\omega }}^{2}\left(t \pm \tau \pm {\alpha }_{\mathrm{PFT}}x\right){E}_{\mathrm{THz}}^{*}\left(t\right)\right|}^{2}\partial t,$$

where *S*_2*ω*_ is the detected PMT signal, ${\chi }^{\left(3\right)}\left(2\omega ,\omega ,\omega ,{\omega }_{\mathrm{THz}}\right)$ is the third-order susceptibility tensor of air within the plasma, ${E}_{\omega }\left(t \pm\uptau \pm{\alpha }_{\mathrm{PFT}}x\right)$ is the electric field of the probe beam delayed by a temporal factor *τ* and influenced by a PFT *α*_PFT_ in s/m, *x* is the transverse coordinate, and ${E}_{\mathrm{THz}}\left(t\right)$ is the THz field confined to the plasma filament. In the PFT system, the probe field is modified as $E\left(x,t\right) \to E(x,t\pm {\alpha }_{\mathrm{PFT}}x)$.

**Fig. 41:**
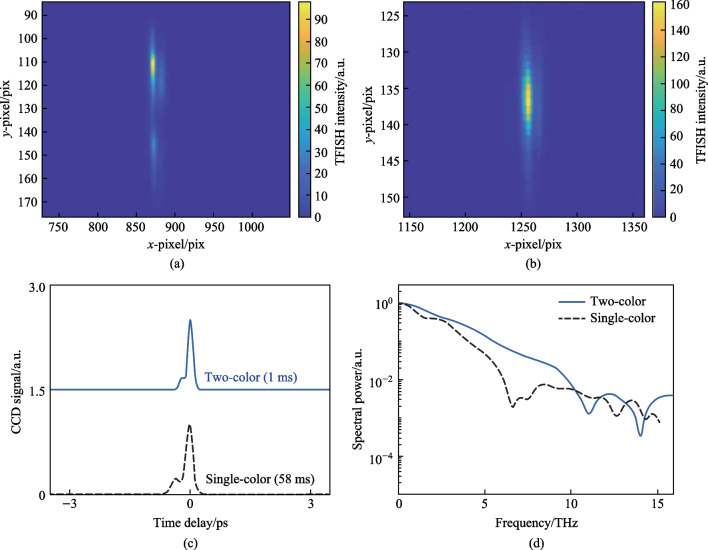
Real-time detection. **a** SH image gathered at the CCD for a single-color pumped signal. **b** Single-shot SH image gathered at the CCD for a two-color pumped signal. **c** Temporal TFISH signals gathered for two-color (solid line) and single-color *ω* (dashed line) pumps resulting from integrating over the row of pixels. The waveforms are flipped along the temporal axis to match previous results. The waveforms are vertically shifted for clarity. **d** Spectra corresponding to the TFISH signals

The real-time trace of the TFISH signal for a single-color excitation pump plasma is shown in Fig. 41a by removing BBO 1. The single-color signal is weak and requires a 1.2 W probe beam power at 1 kHz (1.2 mJ) to produce a visible trace. The exposure time on the CCD must also be increased to 60 ms (resulting in an average over 60 pulses) which makes this measurement decidedly multi-shot. However, the result is a real-time trace where no scanning of the delay is needed outside of the initial system calibration, and the measurement can be done in single shot if a pulse energy of $1.2 \times J*\sqrt{60}=9.3 \, \mathrm{ mJ}$ is used. The required energy for single-shot operation can also be significantly reduced but at the expense of the time window by decreasing the probe beam diameter. If the probe diameter is decreased and if the pulse-front tilt (PFT) is increased, for example by reducing the focal length of the final lens in the grating-imaging system, a higher intensity can be reached and the TFISH signal can be seen easier. Unfortunately, while decreasing the probe diameter has no effect on the spectrometer resolution, increasing the PFT angle will reduce the resolution. The reduction in resolution will be like performing a multi-shot scan of the signal with a delay line at very sparse sampling points.

In Fig. 41b, the TFISH trace is shown for a two-color pumped plasma. The requirement for this signal is an optical probe beam power < 130 mW (< 130 µJ) and appears visible on the CCD with an exposure time of 1 ms. Note that while the required probe energy is much higher than that needed to observe a strong and visible signal in the multi-shot systems shown in Sects. 2 and 3, it is still on the order of the typically used probe energies in conventional TFISH and ABCD measurements [37, 39]. The TFISH signal is very sensitive to P-Scan position and localization of the imaging plane is very important in retrieving the correct signal. However, for a 1 kHz laser with 1 ms exposure, this signal is effectively a single-shot signal and requires no averaging. There are also no moving parts nor is a delay scan required. Both traces in Fig. 41a, b are cropped so that only a small region is shown—because the TFISH process can only occur when the probe interacts with a THz field, and because we surmise that the THz is spatially confined to a small region in the plasma filament, there is little to no background found in the measurement.

To retrieve the temporal trace, an average is performed over every row of pixels in the corresponding images. The CCD used in the experiment has a 2.2 µm pixel size. Experimentally, the PFT method shows a resolution of 22 fs/pix compared to a predicted 20 fs/pix resolution. This resolution is experimentally measured by gathering the single-shot signals at two different delay stage positions. The resolution in fs/pix is the ratio of the time difference between the two stage positions over the number of pixels that the peak of the signal travels between the two stage positions. It should be noted that the spectrometer resolution is different from the real resolution and a 22 fs/pix quantity does not indicate resolving power below the probe beam pulse duration. A linear systems approach reveals that the true resolution of the system is found by analyzing the convolution between the probe optical pulse and Gaussian spectrometer function with its width set to the spectrometer resolution. This analysis shows a true resolution near the probe beam pulse duration. Because the PFT method writes the temporal information directly onto the spatial width of the probe beam, a very large time window is accessible. In a conventional TFISH experiment, the time window can be increased arbitrarily by controlling the beam diameters (probe and THz) and the PFT angle.

The recovered TFISH traces for a single-color and two-color source are shown in Fig. 41c at an exposure of 58 and 1 ms, respectively. The two-color trace is visible to the human eye in a dark room. The corresponding power spectrum is shown in Fig. 41d by performing a Fourier transform operation on the temporal trace. In the above figures, because the noncollinear TFISH system can only produce a signal in accordance with Eq. (30), the process is inherently background-free. However, the appearance of other processes leading to SH radiation secondary to the TFISH signal and optical scattering effects can lead to the appearance of DC components in the TFISH spectrum. The spatio-temporally coupled probe is then modulated by the THz field spatially to represent the TFISH profile temporally. Although the same system as in Sect. 3 is used, Fig. 41c shows that the phase reversal point of the temporal TFISH signal is well defined. This is due to the stringent requirement on the correct imaging position as imposed by the PFT shown simulated in Fig. 40 and to the number of measurement points given by the PFT resolution expression. Additionally, because of the strong angular dispersion and spatial chirp, the interaction length is limited compared to the multi-shot 40° system which leads to less signals being mixed to occlude the phase reversal point. Unfortunately, the nature of STC makes it very difficult to achieve a high contrast in the experiment.

In Fig. 41c, the maximum achievable time window is within the theoretical expectation of 15 ps, but the limited size of the THz beam imposes a limit to the single shot trace. In the case above, the maximum time window achieved is 1 ps. While this time window is relatively short, the PFT TFISH method can be expanded to systems where the THz diameter is larger so that the time window rapidly scales. For example, in a conventional TFISH experiment, a 15 ps time window can be achieved with only a 2 mm diameter focused THz beam.

### Results and discussion: single-shot coherent detection of air plasma sources

Next, the coherent signal for the two-color source is found in the PFT system. The interferometric mixing of the TFISH signal with a controlled second harmonic then provides a coherent THz signal. Using the BBO 2 crystal, the 2*ω* (400 nm) probe is termed a Local Field-Induced Second Harmonic (LFISH) generated wave. Considering that some of the *ω* probe beam propagates unconverted, the interferometric mixing of the two SH signals has the form:

31 $${S}_{2\omega } \propto {\left|{E}_{2\omega }^{\mathrm{LFISH}} + {E}_{2\omega }^{\mathrm{TFISH}}\right|}^{2}.$$

**Fig. 42:**
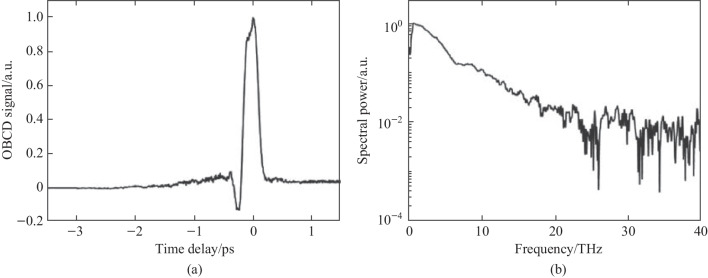
**a** Single-shot OBCD signal from the PFT configuration gathered at 1 ms exposure time. **b** Corresponding Spectral power of **a** obtained through mathematical Fourier transform

The evaluation of the above expression produces two incoherent intensity traces and a cross-correlated term known as the OBCD term. A background subtraction operation is required to extract the OBCD term and omit the LFISH intensity term. The OBCD trace for a two-color source is shown in Fig. 42a along with its Fourier transform in Fig. 42b. In this case, probe instabilities clearly outline the time window as limited by the width of the THz beam (given that it is smaller than the horizontal width of the probe). The PFT coherent detection is achieved with a 1 ms exposure time.

Unfortunately, the THz spectrum can also be limited due to alignment constraints. In the experiments, the BBO 2 crystal has a small aperture and part of the PFT trace can be clipped. Because the BBO must be placed in a region of dominant spatial chirp to avoid damage to the crystal, part of the optical probe spectrum will be cut. When the PFT is established at the interaction region, the clipped trace results in an artificial lengthening of the minimized pulse as predicted by the time-bandwidth product. Because the beam is spatio-temporally coupled, this means that the signal becomes slightly distorted, and the maximum detection spectrum is decreased proportional to the increase in the probe pulse duration. Prior to the interaction region, the temporal information is not localized linearly along the probe beam as expected and due to the angular dispersion, clipping the edges of the beam could lead to reduced information in the overall signal. Despite these effects, the recovered spectrum boasts detection beyond what is possible with single-shot EOS.

**Fig. 43:**
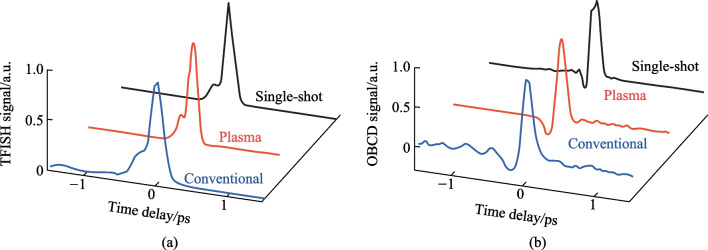
Comparison of **a** TFISH signals and **b** OBCD signals for three systems: (1) the blue line shows the signal from the conventional TFISH/OBCD system as shown in Fig. 3, (2) the red line shows the signals from the plasma-based system as shown in Sects. 2 and 3, and (3) the black lines show the signals from the single-shot system described in this section

In Fig. 43, the single-shot two-color pumped signals are plotted for comparison with the conventional TFISH system. A TFISH/OBCD system like that shown in Fig. 3 was built by guiding the THz from the plasma with the use of 90° off-axis parabolic mirrors (OAPM) and using a probe beam to combine with the collected far-field THz radiation. Using EOS, the far-field THz peak electric field strength was measured to be 130 kV/cm when using a 150 mm focusing OAPM. In Fig. 43a, the TFISH signals for the conventional TFISH system, the plasma-based system in Sect. 3, and the single-shot TFISH system are plotted together. As can be seen, the width of the traces is very similar and the TFISH signals agree very well in their shape. Because the THz intensity pulse durations agree very well—especially between the plasma and single-shot system—the effect of windowing due to the limited 1 ps temporal window are neglected. In Fig. 43b, the THz electric field profiles are measured using OBCD techniques. In the conventional OBCD system, the OBCD signal is gathered by placing the BBO 2 prior to focusing the probe beam as well. The three signals again show good agreement in their profile and duration.

**Fig. 44:**
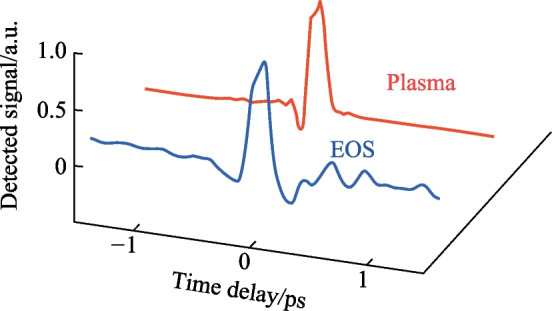
Comparison of single-shot EOS and single-shot plasma OBCD signals gathered while characterizing the same plasma source

Additionally, like the multi-shot case, the SNR, DR, and detection bandwidth can be compared to a single-shot EOS system as seen in Fig. 44. A PFT-based single-shot EOS system as described in Ref. [53] was built to characterize the same plasma source. The crystal used was a 1 mm thick ZnTe crystal and the probe grating was a 1200 lp/mm grating providing a comparable temporal resolution to the plasma system but at a collinear matching between the THz and the optical probe. The EOS system used the same camera as the single-shot plasma OBCD system as well.

The process to experimentally determine the SNR of the plasma system is like the multi-shot case. To estimate the standard deviation, three or more images are taken at a given temporal delay under the same configuration. The standard deviation of the maximum signal across all images is the signal noise for SNR measurement. Finally, the pixel values located at a spatio-temporal position > 10 ps away for each image are taken as the background noise for DR measurement. As seen in Table 2, the SNR and DR for both systems are comparable. However, because of the limited detection on the ZnTe crystal, the plasma OBCD is clearly superior in detecting high spectral frequency components. Additionally, because the EOS system is based on a second-order nonlinear process, the required energy of the probe is four orders of magnitude lower than needed for the plasma system.

**Table 2:** Comparison of system parameters for a single-shot EOS and the single-shot OBCD applied to the same plasma source

System	EO sampling	This work
SNR	~ 200	~ 200
DR	> 10^3^	> 10^3^
Bandwidth	2.5 THz	> 20 THz
Probe energy required	< 10 nJ	< 130 µJ

### Summary

In summary, the local measurement of THz intensity profile and electric field profile inside a plasma by mixing a spatio-temporally coupled probe beam directly into the plasma was presented. The resulting trace is visible to the human eye and serves as a powerful diagnostic of THz radiation in plasma-based sources. A broad detection bandwidth is recovered for a two-color air-plasma THz source. Although limited by the spatial extent of the THz confined to the filament, the methods presented in this section can be extended to conventional TFISH/OBCD/ABCD systems if the peak THz electric field is increased or if a more sensitive CCD is used for detection. Thus, the concepts presented in this section show that the techniques commonly used for single-shot EOS can be readily applied to TFISH/OBCD to develop new detection schemes for large-scale laser facilities.

## Data Availability

Data underlying the results presented in this paper are not publicly available at this time but may be obtained from the authors upon reasonable request.
